# Heterogeneous and higher-order cortical connectivity undergirds efficient, robust, and reliable neural codes

**DOI:** 10.1016/j.isci.2024.111585

**Published:** 2024-12-12

**Authors:** Daniela Egas Santander, Christoph Pokorny, András Ecker, Jānis Lazovskis, Matteo Santoro, Jason P. Smith, Kathryn Hess, Ran Levi, Michael W. Reimann

**Affiliations:** 1 Blue Brain Project, École Polytechnique Fédérale de Lausanne (EPFL), Campus Biotech, 1202 6 Geneva, Switzerland; 2 Riga Business School, Riga Technical University, 1010 Riga, Latvia; 3 Scuola Internazionale Superiore di Studi Avanzati (SISSA), 34136 Trieste, Italy; 4 Department of Mathematics, Nottingham Trent University, Nottingham NG1 4FQ, UK; 5 UPHESS, BMI, École Polytechnique Fédérale de Lausanne (EPFL), 1015 Lausanne, Switzerland; 6 Department of Mathematics, University of Aberdeen, Aberdeen AB24 3UE, UK

**Keywords:** biological sciences, computer science, natural sciences

## Abstract

We hypothesized that the heterogeneous architecture of biological neural networks provides a substrate to regulate the well-known tradeoff between robustness and efficiency, thereby allowing different subpopulations of the same network to optimize for different objectives. To distinguish between subpopulations, we developed a metric based on the mathematical theory of simplicial complexes that captures the complexity of their connectivity by contrasting its higher-order structure to a random control and confirmed its relevance in several openly available connectomes. Using a biologically detailed cortical model and an electron microscopic dataset, we showed that subpopulations with low simplicial complexity exhibit efficient activity. Conversely, subpopulations of high simplicial complexity play a supporting role in boosting the reliability of the network as a whole, softening the robustness-efficiency tradeoff. Crucially, we found that both types of subpopulations can and do coexist within a single connectome in biological neural networks, due to the heterogeneity of their connectivity.

## Highlights

• A topology-based metric captures network complexity and distinguishes subpopulations
• Subpopulations with low simplicial complexity drive efficient activity
• Subpopulations with high simplicial complexity support network reliability
• Both can and do coexist within a single connectome in biological neural networks

## Graphical abstract

**Figure:**
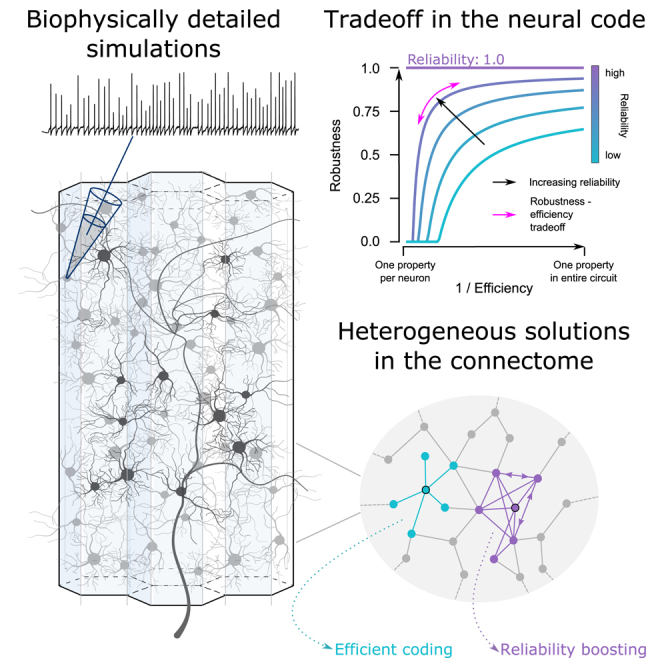

## Introduction

Neuronal activity is typically unreliable, i.e., highly variable across repetitions.^1^^,^^2^^,^^3^ While part of this variability may have a functional role, some of it is simply noise coming from a wide variety of sources, such as synaptic failure.^4^^,^^5^^,^^6^ This variability poses a fundamental problem for a robust, i.e., error-resistant, code. One proposed solution is population coding, i.e., encoding the same information across multiple neurons, e.g., through neuronal assemblies.^7^^,^^8^^,^^9^ If the activity of the neuronal population is highly correlated and its activity manifold,^10^ therefore, low dimensional, the added redundancy provides a population code that can overcome noise and thus encode information robustly. On the other hand, if the activity is mostly uncorrelated and its activity manifold, therefore, high dimensional, the information is encoded efficiently.^11^^,^^12^^,^^13^^,^^14^^,^^15^^,^^16^

**Figure 1:**
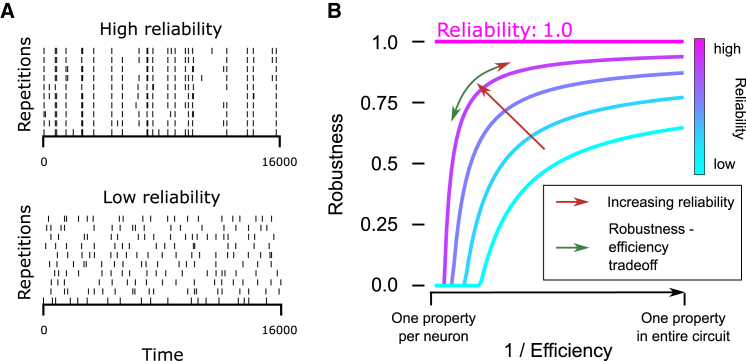
The efficiency-robustness tradeoff (A) Examples of neurons with low/high reliability. (B) There is a tradeoff between efficiency and robustness in a neuronal circuit. The severity of the tradeoff is mediated by the level of neuronal reliability.

Conceptually, thus, circuits evolve to optimize the tradeoff between *robustness* and *efficiency*, mediated by the degree of neuronal *reliability*. We explain this interaction with the following simple thought experiment. Consider a neural circuit in a sensory region. Assume neurons have a given level of reliability, which measures how similar their responses are across repetitions of a stimulus (Figure 1A). Note that, for the purpose of this illustration, we make no claim about the source of variability, e.g., noise or non-stimulus-related information. If the neurons were perfectly reliable, a number of (uncorrelated) properties up to the number of neurons could be encoded without errors, i.e., at maximal robustness (Figure 1B, pink). For less-than-perfect reliability, this number of properties can be encoded only at the price of a loss of robustness. Alternatively, the level of robustness can be maintained by reducing the number of properties encoded (Figure 1B, purple). The severity of the tradeoff can be reduced by increasing neuron reliability, thereby shifting to a more favorable curve in the plot.

Note that related notions of efficient coding have been formulated and mathematically investigated before, two of which appear frequently in the literature: sparse coding^17^^,^^18^ and maximal network capacity.^19^^,^^20^^,^^21^ Though based on different mathematical foundations, information theory and dynamical systems, respectively, these two notions are not in conflict with our definition. Moreover, they also confront an efficiency-robustness compromise exhibited as a tradeoff between minimizing the number of active neurons while remaining “overcomplete” in the first case and maximizing both the number of basins of attraction and the distance between them in the second. In both cases, the severity of the tradeoff is mediated by a certain level of amplitude of a noise term playing the role of neuronal reliability. Furthermore, on a related note but from the perspective of complex dynamical systems, the question of what optimizes computational capacity has been explored.^22^^,^^23^^,^^24^^,^^25^ In the discussion, we further examine how their concept of “computing at the edge of chaos” or “self-organized criticality” connects to our work.

The main purpose of this work is to study how the efficiency-robustness tradeoff plays out in neural circuits. Is it uniform between subnetworks, such as layers? Given that synaptic connectivity shapes the neural code, how does its structure affect the outcome of the tradeoff? Beyond this tradeoff (Figure 1B, green arrow), increasing reliability globally is a way of improving robustness of the code without loss of efficiency (Figure 1B, red arrow). Can we find evidence of connectivity mechanisms enhancing reliability of firing? If so, are they employed equally, or do they favor some subpopulations?

As our goal involves linking the structure of connectivity to a functional measure, we need relevant network metrics, as well as structural data together with co-registered functional data. Higher levels of functional redundancy have been achieved by more tightly interconnecting a subpopulation.^26^^,^^27^^,^^28^^,^^29^ Thus, we employ a mathematical framework based on finding densely connected motifs called *directed simplices* and describing how they are connected to each other to form larger motifs. A directed simplex of dimension *k* is a motif of k+1 neurons all-to-all connected in feedforward fashion (see Box 1). Simplices have been demonstrated to have functional relevance.^30^ In particular, they are related to the reduction of dimensionality of neural activity and the formation of assemblies, as the correlation of spiking activity of neurons increases with the dimension of simplices they participate in.^31^^,^^32^ Structurally, they have been shown to be overexpressed motifs in neural networks at all scales,^33^^,^^34^^,^^35^^,^^36^^,^^37^ even though constructing random sparse directed graphs with a specified overexpression of simplices is an unsolved mathematical problem.^38^^,^^39^ Finally, this framework offers great analytical flexibility as it provides a range of network metrics that describe not only the global structure of a graph but also its local structure, i.e., node- or even edge-based metrics. These node-based metrics are generally strongly related to other notions of node centrality obtained from pairwise interactions.^33^^,^^40^ Thus, they provide a complementary perspective on other metrics describing higher-order structure, such as rich clubs, clustering, or small-world coefficients.^41^^,^^42^^,^^43^^,^^44^

Box 1 **Simplices in directed networks**

**Figure 2:**
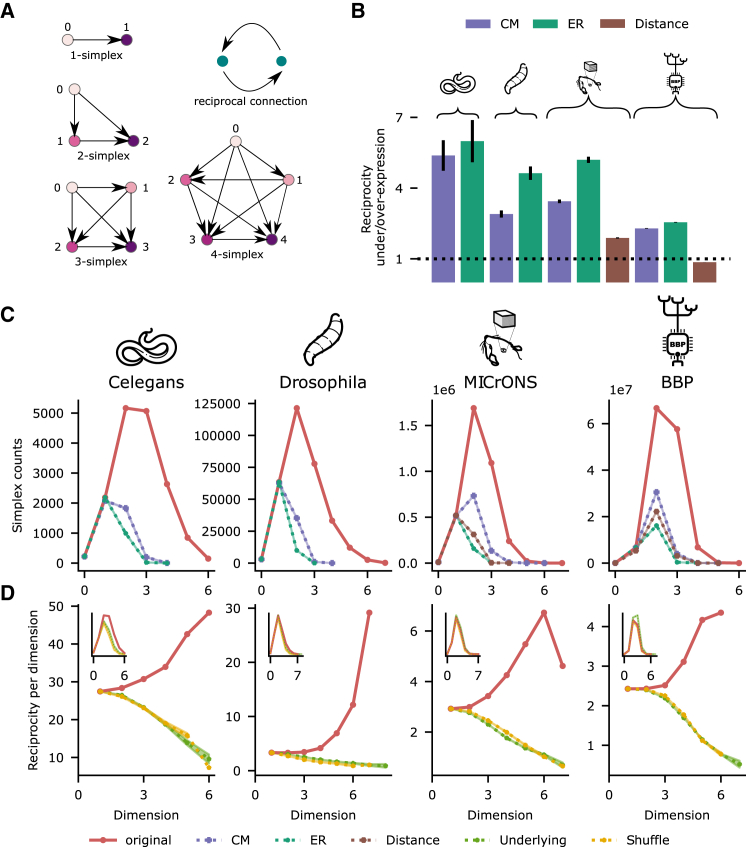
Overexpression of directed motifs (A) Reciprocal connection (green nodes) and simplices (purple nodes). (B) Percentage of reciprocal connections in each connectome relative to mean of the percentage of 100 controls of different types: Erdős–Rényi, configuration model, and distance dependent (see STAR Methods). A value of 1 indicates the original connectome has the same percentage of reciprocal connections than its corresponding control; values above/below 1 represent over/under expression; vertical bars show the standard deviation. All values are significantly different than the mean with *p* values under $3\times 10^{-155}$ for a two-sided one-sample t test. (C) Overexpression of simplex motifs with respect to 10 random controls, which by design match the counts of the original network for dimensions 0 and 1 (see STAR Methods). Dots denote mean values in that dimension and shaded regions are the standard error of the mean. All counts in dimensions greater than one are significantly higher than the mean of the controls with *p* values under $4\times 10^{-18}$ for a one-sided one-sample t test. (D) Percentage of reciprocal connections in the subgraph of simplices of each dimension contrasted with the same curve for controls where just the directionality of the connections is modified (see STAR Methods). Dots denote mean values in that dimension and shaded regions are the standard error of the mean. Inset: simplex counts of the directional controls are close to the original ones.

A directed simplex of dimension *k* is a motif of k+1 neurons where the connectivity between them satisfies the following criterion: there exists a numbering of neurons in the motif from 0 to *k*, such that if i<j then there exists a synaptic connection from $n_{i}$ to $n_{j}$ (Figure 2A). We call neuron $n_{0}$ the *source* and neuron $n_{k}$ the *sink* of the simplex.

As the functional properties central to this work, i.e., efficiency and robustness, are population based, we need population-based structural metrics that we can link to them in addition to the node-based ones. Thus, we developed a population-based structural metric that is also related to simplex motifs, which we call *simplicial neighborhood complexity*. Briefly, it compares the synaptic connectivity of a subpopulation to that of a random control.

To demonstrate the usefulness of simplex-based metrics, we first show that they capture non-random trends in many different biological neural networks and that they are related to previously studied phenomena, such as overexpression of reciprocal connections.^45^^,^^46^ To benchmark our structural metrics, we analyzed several networks with cellular resolution: the mature *C. elegans*,^47^ *Drosophila* larva,^48^ and an electron microscopic reconstruction of mouse cortical connectivity^49^ (MICrONS). Note that, while the MICrONS dataset is imperfect, the error rate of this reconstruction has been determined, and we show that the results of our analyses cannot be explained by errors at that rate. Additionally, the MICrONS dataset also provides co-registered activity, which we can use to study the structure-function relation. However, the co-registered activity data in MICrONS are too sparse to estimate some population-based functional metrics. Thus, we additionally generated activity data from simulations of a morphologically detailed model^50^ (BBP) to confirm the relevance of the metrics for reliability, robustness, and efficiency and to develop a detailed picture of the structure-function relation.

We believe that the BBP network is relevant for our use case as it provides full certainty and control of both structural and functional results. It enables more complex analyses and connectivity manipulations that allow us to link the structure of connectivity to function in more detail and, in some cases, in causal ways. Further, it captures much of the biological detail that is relevant for our field of enquiry, such as sources of variability, in particular, synaptic failure, spontaneous release, and stochastic ion channels. Extrinsic input from other cortical regions is modeled with a relatively simple noise model. Additionally, the BBP model has already been used to study the question of noise and firing variability.^30^ Finally, we will demonstrate that the BBP network reproduces the same non-random simplex-based structural features that are present in biological neural networks. While they emerge less strongly in the model, this is nevertheless highly significant, as we will demonstrate that the features cannot emerge by chance. We will also demonstrate that they correlate with measures of efficiency and reliability in qualitatively the same way as in biological neural networks.

With these tools and data sources, the following are our main results. First, we show that the greater the number of simplex motifs to which a neuron belongs, the higher the correlation of its activity with that of the population as a whole. This leads to coding in subnetworks with higher density of simplex motifs being less efficient, but generally more robust, demonstrating the impact of simplex motifs in positioning the cortical code along the robustness-efficiency tradeoff (Figure 1 green arrow). In particular, the highest level of simplex overexpression leads to low efficiency with little to no increase in robustness. Then, we show that the presence of such highly complex subnetworks globally increases spiking reliability of neurons (Figure 1 red arrow). Further, we show that the increase in reliability through higher density of simplex motifs and reciprocal connections also affects reliability at the single neuron level, albeit weakly.

## Results

### The overexpression of reciprocity is shaped by the higher-order structure

To confirm previous results about the structural relevance of reciprocal connectivity and simplex motifs (see Box 1), we counted their overexpression in the following four available connectomes at cellular resolution: first, the electron microscopy reconstructions of the full brain connectivities of the adult *C. elegans*^47^ and the *Drosophila larva*^48^; second, the electron microscopy reconstructions of 1 mm^3^ volume of mouse primary visual cortex (*MICrONS*^49^) and a morphologically detailed model of ∼2 mm^3^ of rat somatosensory cortex (*BBP*^50^). See STAR Methods for details on the data sources for all connectomes.

Across all connectomes, we found an overexpression of reciprocal connections and directed simplex counts when contrasted to relevant control models of the same size (Figures 2B and 2C). The controls considered were *Erdős–Rényi* controls, *directed configuration model* controls (CM), and *distance dependent* controls, which preserve the overall density of connections, the in and out degrees of the nodes, and the density of connections at a given distance, respectively (see STAR Methods and Table 1). Note, in particular, that the CM controls will preserve the known long-tailed degree distributions.^51^^,^^52^^,^^53^^,^^54^ The distance-dependent controls were only performed for MICrONS and BBP, given that Euclidean distances between cell bodies might not be the most relevant factor determining connection probability for *C. elegans* and *Drosophila*. Overexpression of reciprocal connections was weakest in the BBP model connectome, to a degree where it was not overexpressed compared to the distance-dependent control. All other overexpressions of reciprocal connections and simplex counts were statistically significant at the most stringent significance levels. In particular, this shows that distance-dependent connectivity and degree heterogeneity contribute but do not fully explain the high complexity of the biological connectivity structure across species. This is unlike in simple network models, optimized for information storage, where higher-order structure can be explained from a wide-degree distribution.^46^

**Table 1:** Types of random graphs

Label	Model name	Property maintained
ER	Erdős–Rényi	overall density of connections
CM	directed configuration model	sequence of in-/out-degrees
Dist	distance dependent	density of connection at a given distance
Und	underlying	underlying undirected graph
rcSh	rc-shuffled	subgraph of non-reciprocal connections

The lower a model is in the table, the closer it is to the original graph.

In addition to simplex counts as a global metric of network complexity,^55^^,^^56^^,^^57^ we can quantify the participation of individual elements in that structure. For example, *k-node participation*, defined as the number of *k*-simplices to which a node belongs, provides a generalized notion of node centrality highlighting the relation between simplex counts and more classical metrics in network science. Indeed, we confirmed that in- and out-hub nodes have higher node participation than the rest of the population (Figure S1). Moreover, node participation is correlated to closeness centrality (Figure S2), a metric measuring how close a node is to all other nodes in the graph in terms of path distance.^58^ Similar observations, relating simplex counts and other centrality metrics, have been made before for other biological networks.^33^^,^^40^

On the other hand, *k-edge participation*, defined as the number of *k*-simplices an edge is part of, gives a notion of edge centrality that has been observed to be meaningful for describing synaptic strength and predicting plastic changes, in both MICrONS and the BBP connectome.^59^ We can filter edges by the maximum dimension at which this metric is non-zero. Doing this, we found that edges with a high maximum dimension are more likely to be reciprocal, and this preference increases with dimension (Figure 2D, red curves). Moreover, this preference is not merely a combinatorial artifact. To show this, we contrasted these curves with those obtained from two types of random controls: one (*rc-Sh*) only modifies the location of reciprocal connections and the other (*Und*) modifies their location together with the direction of all the connections (see STAR Methods). Crucially, these controls are quite stringent in that they have the same underlying undirected graph as the original and thus approximately preserve the number of simplices across dimensions (Figure 2D, inset). We observed that the percentage of reciprocal connections in these controls actually decreases with dimension, showing that, combinatorially, it is less likely for a reciprocal connection to exist on higher dimensional maximal simplices if these were placed at random.

Note that we studied only a central subvolume of the MICrONS connectome, in order to avoid any boundary artifacts, as well as to have a cylindrical volume comparable to the BBP connectome. Furthermore, synaptic contacts for the MICrONS connectome were automatically segmented, and their corresponding partners were automatically assigned. It has been estimated and reported that the automated detection has a: “precision of 96% and recall of 89% with a partner assignment accuracy of 98%.”^49^ We therefore generated controls to address all these potential sources of error by removing 4% of the connections, adding (1/0.89)−1=12% of connections, and shuffling 2% of the connections, taking into account precision, recall, and partner accuracy, respectively. We observed that simplex counts and their relation with reciprocal connections remained unchanged (Figure S3), showing that our metrics are relevant even at the levels of reported inaccuracy of this data source.

In summary, we confirmed previous results that reciprocal connections (a circular motif) and simplices (a feedforward motif) are strongly overexpressed in biological neural networks (BNNs) and that their locations within the networks are strongly related. These trends also hold true for the BBP network. Moreover, these metrics allowed us to show that the complexity of BNNs cannot be explained by only distance-dependent connectivity, long-tailed degree distributions, or connectome reconstruction errors.

### Biological neural networks exhibit a large diversity of neighborhoods

**Figure 3:**
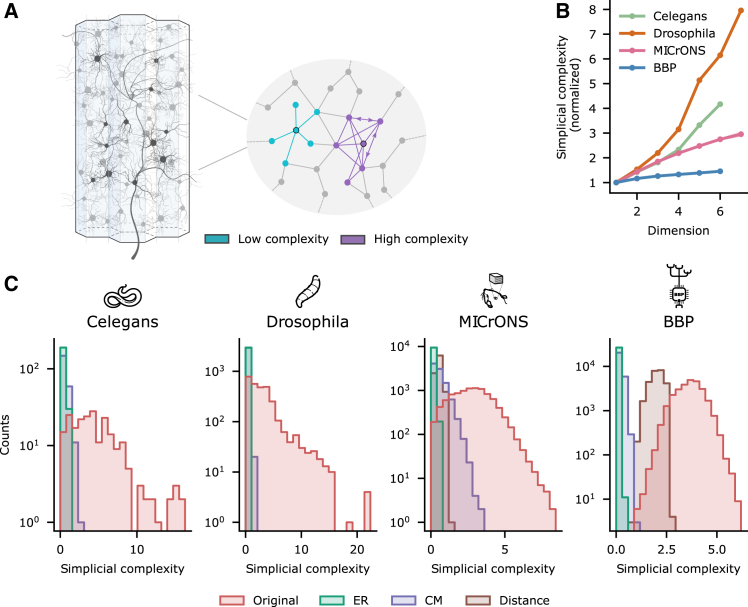
Neighborhood complexity in BNNs (A) Illustration showing a connectome and two neighborhoods of high (purple) and low (blue) complexity. (B) Average of simplicial neighborhood complexity on nodes that are on simplices of a given dimension (see STAR Methods) normalized so that they all have complexity 1 on the first dimension. (C) BNNs have longer tailed distributions of neighborhood complexity than relevant controls (described in Figure 2C).

In the previous section, we showed that BNNs exhibit non-random global structure. It turns out that individual neurons participate in diverse ways in this structure, which could affect their function. We make this clear in two complementary ways. First, on the level of single neurons, we exploit the *simplicial structure*, taking averages of functional or structural properties of neurons weighted by the number of maximal simplices in which they participate (see STAR Methods). This analysis reveals the effect of the simplicial centrality of a neuron on its functional properties. Second, on the level subpopulations, we analyze the synaptic *neighborhood structure* of a neuron, given by all neurons directly interacting synaptically with a central one. We define the *neighborhood* of a neuron *v* as the subnetwork on *v*, together with all neurons connected to it (in either direction) and all connections between them, and call *v* its *center* (Figure 3A). One can then apply any network metric to neighborhoods, a particularly simple example being the *neighborhood size*, i.e., the number of constituent nodes, which closely approximates the sum of the in- and out-degrees of its center (see STAR Methods). As expected from previous research,^41^^,^^53^^,^^54^^,^^60^ we found across connectomes that the distribution of neighborhood sizes has a longer tail than in corresponding controls, except for CM controls, which fix the node degrees and thus the neighborhood sizes. However, for a plethora of other network metrics, the distributions of their values are also more long tailed than all corresponding controls (see Figure S4 for examples) including CM, indicating that this difference is not driven by long-tailed node degree distributions alone.

This suggests that the neighborhoods of a BNN exhibit a wide range of complexity, which may fulfill different functional roles. To test this hypothesis, we need to be able to capture this complexity in a single number independent of the neighborhood size. We propose a metric of neighborhood complexity based on its distance to a random network of the same size in terms of normalized simplex counts and call this *simplicial neighborhood complexity* (see STAR Methods and Figure S5 for an explicit example). We will often refer to this measure as *neighborhood complexity* in short, to ease readability. The distribution of its values is also long-tailed with respect to controls (Figure 3C). We computed the average neighborhood complexity across maximal simplices and found that it increases with dimension for all connectomes (Figure 3B), indicating that the simplicial structure and neighborhood complexity are strongly related Additionally, simplicial neighborhood complexity is related to the density of reciprocal connections in the neighborhood (Figure S6C) as well as to an alternative measure of neighborhood complexity based on distances of degree distributions instead of simplex counts (see STAR Methods; Figure S6D). The latter confirms the robustness of our metric.

In summary, a structurally highly diverse set of local subnetworks (i.e., neighborhoods) together form the non-random global structure of BNNs. Moreover, the complexity of neighborhoods can be measured by a variety of metrics that are related to each other but complementary. We chose simplex counts as our main metric, as it captures the complexity of the global structure of BNNs and has demonstrated functional relevance.

### Low-complexity neighborhoods are efficient

So far, we have shown that BNNs exhibit a wide range of neighborhood complexities, as measured by a variety of network metrics. We hypothesize that this heterogeneity provides a substrate for the diversity observed in neuronal function related to the robustness-efficiency tradeoff. More precisely, prior work has shown that the spike correlation between synaptically connected neurons increases with the dimension of the largest simplex of which the connection forms an edge.^31^^,^^49^ We therefore hypothesized that on the single-cell level, the correlation of the spike train of a neuron with the overall activity of the circuit increases with the number of simplices to which it belongs. On the subpopulation level, neurons belonging to a neighborhood of high simplicial complexity should exhibit more highly correlated activity among themselves. Both effects would reduce the efficiency of the code.

We tested these hypotheses for the MICrONS and BBP connectomes, which have co-registered structural and functional data. For MICrONS, calcium traces of a sparse, subset of neurons (up to 2.5% per scan and up to 20% when pooled across scans; see STAR Methods) were reported for five classes of naturalistic stimuli^49^ (see STAR Methods; Figure S7A). For BBP, the spike trains of all neurons were reported for eight repeated stimuli (see STAR Methods; Figure S7B).

**Figure 4:**
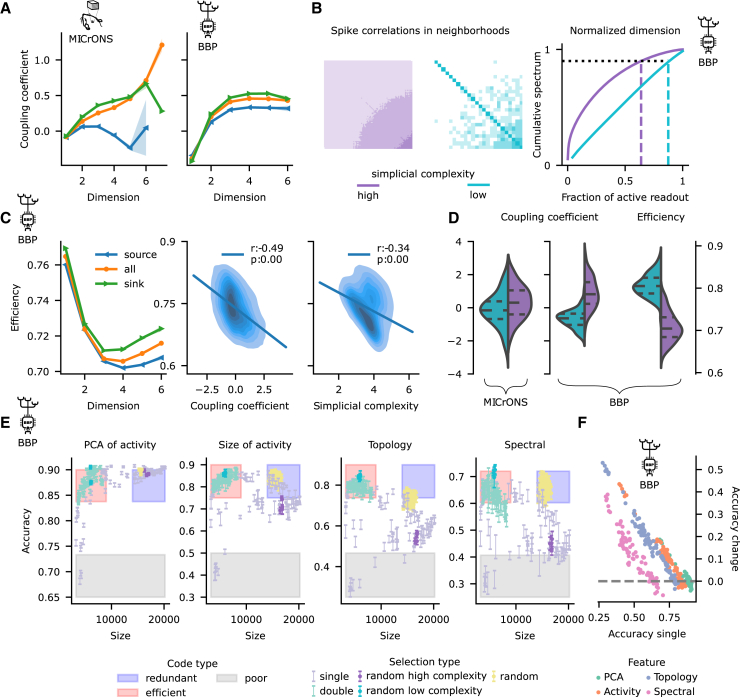
Low-complexity neighborhoods are more efficient (A) Coupling coefficient (*Z* scored) increases with maximal simplex dimension for MICrONS (left) and BBP (right). Sinks of neurons (green curves) are more coupled than sources (blue) and the overall population (orange). Dots denote mean values and shaded regions are their SEM. (B) Left: correlation matrices of the spike trains of the lowest/highest complexity neighborhoods of the network in the BBP data. Right: their corresponding normalized spectra from which their *efficiency* is determined. (C) Efficiency for the BBP data, from left to right: efficiency decreases with maximal simplex dimension colors and shaded regions as in (A). Efficiency is anti-correlated to the coupling coefficient and simplicial neighborhood complexity. (D) Left: the centers of the 100 least complex neighborhoods have a mean coupling coefficient that is significantly lower than the centers of the 100 most complex ones, with *p* values of $1.66\times 10^{-3}$ and $8.25\times 10^{-28}$ on the Kruskal-Wallis test for MICrONS and BBP, respectively. Right: the opposite effect is observed for efficiency, with a *p* value of $2.60\times 10^{-34}$ (BBP data only). (E) Accuracy of the classification task versus size of the subpopulation used for four different featurization methods. Less complex neighborhoods (light blue) consistently provide higher accuracy with smaller subpopulations, even when these are chosen at random (cyan). (F) The change in accuracy in the double selection process (see STAR Methods) is anti-correlated to the accuracy of single selection, showing that low complexity is a sufficient condition for efficient coding.

To test the first part of the hypothesis, we built on previous work, where a wide spectrum of functional roles was observed experimentally, showing that neurons can differ in their activity correlation to the overall firing of the population, i.e., their *coupling coefficient*, ranging from strongly coupled *choristers* to weakly coupled *soloists*.^61^^,^^62^ We found for both MICrONS and BBP that the simplicial structure shapes the value of the coupling coefficient: neurons that belong to higher dimensional maximal simplices have higher coupling coefficients (Figure 4A). This shows that soloists/choristers are in the lower/higher simplicial dimensions of the connectomes. Moreover, Okun et al.^61^ also found that the coupling coefficient increases with the amount of synaptic input. Conversely, Brunel^46^ used simpler network models to predict that neurons with large out-degree are active in smaller assemblies. Intuitively, this would reduce their coupling coefficient and lead to a higher node participation in the source than in the sink position. By showing that sinks of simplices are more coupled than sources, we confirm these findings and extend them by showing that the effect is even stronger if the presynaptic population is highly recurrent.

In order to test the second part of our hypothesis, we considered the correlation of activity on the level of subpopulations. Previous work pointed out that mostly uncorrelated activity maximizes the amount of information that can be encoded and is thus a mark of an efficient code.^11^^,^^12^^,^^13^^,^^14^^,^^15^^,^^16^ Based on this, we defined the *efficiency* of a neighborhood as the fraction of its neurons required to explain 90% of the variance of its correlation matrix (see STAR Methods, Figure 4B). The sparsity of the MICrONS functional dataset makes it impossible to obtain an estimate of this, or any other metric based on pairwise correlations, that is representative for neighborhoods of that connectome. Thus, we restrict our analysis to the BBP dataset. As we hypothesized, efficiency decreased with the dimension of maximal simplices (Figure 4C-left), and generally, sinks of neurons were more efficient than sources. From a more fine-grained perspective, we found that across layers, neighborhoods centered on sink neurons were also more efficient than neighborhoods of source neurons and that globally neighborhoods with centers in layer 6 were more efficient than other layers (Figure S9C). Moreover, the efficiency of a neighborhood is anti-correlated with its simplicial neighborhood complexity, and highly efficient neighborhoods tend to have soloists at their center (Figure 4C-right). This effect is more pronounced for the *champions*, i.e., the 100 neighborhoods that either minimize or maximize simplicial complexity. Between them, we found a statistically significant difference in their efficiency as well as in their coupling coefficient (Figure 4D-right).

The observed trends should make low-complexity neighborhoods efficient readout populations for information processed in a local circuit as well. We tested this in a simple stimulus classification task. For this, we simulated evoked activity in response to a random stream of the 8 stimuli used before (Figure S7B). Each stimulus was presented 100 times in random order with an inter-stimulus interval of 200 ms. We then tried to determine the stimulus present in a given time window from the spiking activity of a subpopulation of neurons. In order to test the effect of neighborhood complexity, we selected the subpopulations as neighborhoods at different points along the complexity spectrum.

Specifically, we used the neighborhood-based classification pipeline developed in the study by Reimann et al.^63^ and Conceição et al.^64^ Briefly, it selects 50 neighborhoods either at random, or by the ones that maximize or minimize a given network metric from Table S1. We call their original pipeline the *single selection* procedure. This will sample neighborhoods that are either a generic representative of a circuit neighborhood or structural outliers in one way or another. We explicitly consider outliers, as the values of network metrics over neighborhoods are long tailed^53^^,^^54^ (Figure S4) and random sampling may miss points from the extreme ends. Then, the spiking activity of the union of neighborhoods is reduced to a set of time series that we call the *feature vectors*. This featurization is done in two ways: either by applying principal component analysis to the binned spike trains (*PCA method*; bin size 20 ms) or by calculating the value of one of the network metrics listed in Table S1 of the *active subnetwork* of each neighborhood and in each time step (see STAR Methods). That is, we reduce the dimensionality of the spike trains of the subpopulation in different ways to ensure the robustness of our results. Finally, a simple linear classifier is trained to identify the stimulus identity from these feature vectors.

Unsurprisingly, we found that the classification accuracy tended to be higher when the size of the union of the neighborhoods was larger (Figure 4E, purple bars; Figure S10) (This is an example of the tradeoff between robustness [classification accuracy] and the efficiency [the inverse of size] presented in Figure 1B). However, we were primarily interested in efficient coding, not its absolute quality. We considered a neighborhood to be more efficient than another if it reached the same or higher classification accuracy with fewer neurons. As we hypothesized that less complex neighborhoods are more efficient, we executed a *double selection* procedure, where we first selected the 1% least complex neighborhoods and ran the full pipeline described earlier on those neighborhoods only. When considering the size of the neighborhood union against classification accuracy, we found the following: first, double selection resulted in smaller neighborhoods. Second the data points associated with double selection had comparable or better classification accuracy despite the smaller neighborhoods (Figure 4E, light blue and cyan bars, red-shaded region). They were therefore more efficient according to our definition.

The overall level of classification accuracy depended on the network metric used to calculate the feature vectors (Figure S10). For a minority of metrics (9 out of 32), the classification was poor and did not improve for double selection. In these cases, less than ∼4.5% (generally less than ∼2%) of the entries of the feature vectors were nonzero (Figure S11). That is, the featurization was degenerate since virtually no input information was given to the classifier. For some selection parameters, classification accuracy was already high in the single selection paradigm. In those cases, it did not further increase for double selection. Instead, double selection ensured high accuracy where accuracy for single selection was low (Figure 4F). This demonstrates that low complexity is a sufficient condition for efficient coding.

In summary, we confirmed our hypothesis that low/high complexity is associated with low/high-activity correlations. Consequently, neighborhoods of low complexity encode information most efficiently. On the other hand, neighborhoods of high complexity have choristers at their centers that follow the global activity of the circuit. This is an example of the efficiency-robustness tradeoff.

### High-complexity neighborhoods promote reliability

As described earlier, we have shown that the most complex neighborhoods are the least efficient in terms of the amount of information they can encode relative to their size. We have also revealed that the architecture of BNNs contains a diversity of neighborhoods exhibiting long-tailed distributions across network metrics. In particular, they exhibit an overexpression of simplex motifs and a larger abundance of complex neighborhoods than simpler architectures (Figures 2C and S4 bottom), leading us to question the purpose of this overexpression. Given that it has been shown that simplex motifs promote network reliability,^30^^,^^65^ we hypothesized that one of the functions of the highly complex neighborhoods is to promote *reliability* of the network as a whole, improving the robustness-efficiency tradeoff (Figure 1B). In order to test our hypothesis of a global effect, we needed to manipulate the overall connectivity and measure the resulting network’s activity. This can naturally only be done *in silico* and thus we started by studying the effect of connectivity on reliability for the BBP connectome. A neuron is reliable if its firing pattern is consistent (i.e., highly correlated to itself) across several equivalent repetitions. The reliability values were computed as the *Gaussian kernel reliability*^66^ of the spike trains of eight stimuli over ten repetitions (see STAR Methods, and Figure 1A for two exemplary neurons of high and low reliability).

**Figure 5:**
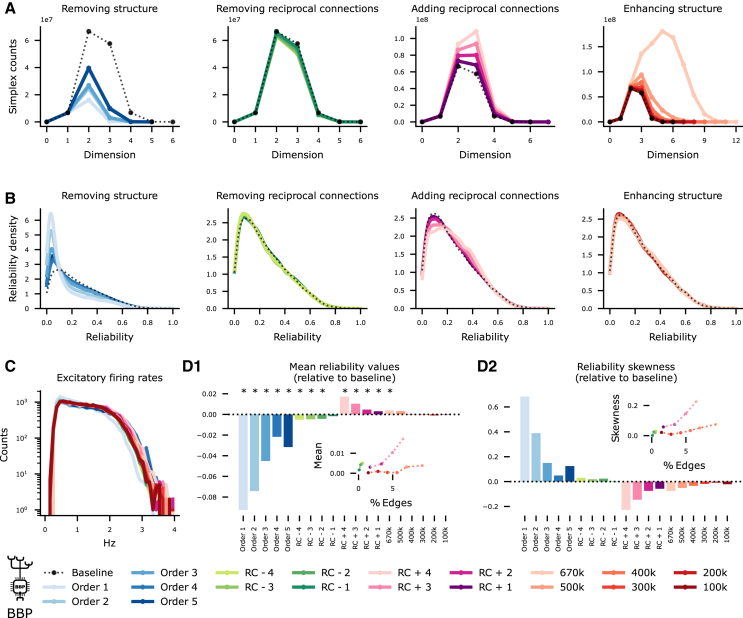
Network complexity enhances network reliability globally (A) Simplex counts of manipulated connectomes. Across all panels, black denotes the baseline values, blue and green reduce complexity, and red and purple increase complexity; lighter tones denote stronger manipulations than darker tones. (B) Distribution of the reliability values of all the neurons in the different manipulated connectomes relative to the baseline. (C) Firing rates remain stable across all manipulations. (D1) Mean reliability values of the manipulated connectomes relative to the baseline. Positive values denote an increase of reliability in the manipulated connectome, the ∗ marks manipulations for which the change on the mean reliability is statistically significant (with *p* values smaller than 0.009 with n=26,567 on the Kruskal-Wallis test). Inset, absolute value of the change of the mean versus the percentage of the edges that have been manipulated. The reduced complexity models are not shown in the inset since virtually 100% of the edges are modified in these manipulations. (D2) As (D1) but for skewness of the distribution rather than the mean value.

We employed the recently developed Connectome-Manipulator Python framework,^67^ which allows rapid connectome manipulations of network models in the SONATA format^68^ (see STAR Methods). We performed manipulations that increase or decrease the network’s complexity in different magnitudes while keeping the overall density constant (see Table 2 and Figure S12), and we use overall simplex counts as a metric for global complexity (Figure 5A).

**Table 2:** Types of connectome manipulations

Label	Modification category	Description
Order1	reduce complexity	uniform probability of connection
Order2	reduce complexity	distance-dependent probability of connection
Order3	reduce complexity	bipolar distance-dependent probability of connection
Order4	reduce complexity	offset-dependent probability of connection
Order5	reduce complexity	position- and offset-dependent probability of connection
RC−1	remove reciprocity	remove reciprocal connections in maximal dimension n≥5
RC−2	remove reciprocity	remove reciprocal connections in maximal dimension n≥5 and 50% at n=4
RC−3	remove reciprocity	remove all reciprocal connections in dimension n≥4
RC−4	remove reciprocity	remove all reciprocal connections
RC∗−K	remove reciprocity	remove the same number of reciprocal connections as in RC−K at random, for 1≤K≤3
RC+K	add reciprocity	increase reciprocal connections on maximal simplices by a factor of K+1, for 1≤K≤4
RC+5	add reciprocity	increase reciprocal connections on maximal simplices by a factor of 8
RC∗+K	add reciprocity	add the same number of connections as in RC+K at random, for 1≤K≤5
Nk	enhance complexity	rewiring of Nk edges to increase edge participation, for *N* = 100, 200, 300, 400, 500, 670

We performed four different types of manipulations that *rewire* the model connectome to change its complexity in ways that are consistent with the structural metrics we have shown to shape BNNs across species. That is, they increase or decrease simplex counts while keeping the number of connections fixed or they remove or add reciprocal connections in simplices (see STAR Methods and Table S1). Moreover, different manipulations rewire a different amount of connections. Note, however, that achieving a given number of simplex counts and reciprocity in unison remains an open problem in combinatorial and stochastic topology.^38^^,^^39^

We found that reducing network complexity always lowered the mean reliability of the network, while the opposite happens for increased complexity (Figure 5A vs. 5B). At the same time, other spiking statistics, such as firing rate distributions, remained unchanged (Figure 5C). In most cases, the change of mean reliability was statistically significant (Figure 5D1, Kruskal-Wallis test for n=26,567), and the *p* value as well as the size of the change was strongly determined by the percentage of the edges that had been rewired (Figure 5D1-inset). On the other hand, even an extreme increase in simplex counts improved mean reliability by less than 1% (Figure 5D1-light orange), indicating that large simplices are not increasing reliability on their own, but are related to a structural effect that increases reliability and thus provide a convenient way to measure it.

Moreover, the number of connections rewired is not the only driver for the change in reliability; the location of this rewiring within the whole network also plays a significant role. In particular, “adding reciprocal connections” has a stronger effect on the network’s reliability than “enhancing structure” even when a similar number of edges is rewired. Furthermore, this is not due to a (slight) increase in overall connection count for the “adding reciprocal connections” manipulation. To demonstrate this, we added or removed the same number of connections as in Figure 5, but instead of doing so in a structured way, we do it at random in the whole excitatory network (see STAR Methods and Table S1). There is a substantial difference in the change in reliability between structured and random manipulations (Figures S13A–S13C). In the extreme case, where we increased by an 8-fold the number of reciprocal connections, the system has a phase transition from asynchronous to synchronous firing regime (Figures S13B2, S13D1, and S13D2). However, in the random control, there is only a moderate increase in the reliability and activity of the network.

Note that even for the most severe reduction of complexity, a small number of highly reliable neurons in a long tail of the distribution remained. Therefore, we tested whether the increase in mean reliability was caused by a global shift or rather by increasing the presence of small populations of highly reliable neurons. This can be captured by the skewness of the reliability distributions, which intuitively is given by the normalized difference between the mean and the median (see STAR Methods). A unimodal distribution has a skewness of 0 if it is symmetric and above/below 0 if it is skewed to the right/left. We found that increasing network complexity decreases the skewness of the reliability distribution (Figure 5D2), showing that the mean reliability increase is due to a global network effect.

Having established that network complexity increases the reliability of the network globally, we next studied its potential local effect on neuronal reliability, i.e., on the level of single neurons and neighborhoods. Given that testing local effects does not require connectivity manipulations, we analyzed this for both the MICrONS and BBP connectomes. For MICrONS, reliabilities were provided by the authors of the original publication, for six fixed stimuli that were each presented ten times, as the *Oracle score* of the deconvolved calcium traces over repetitions (see STAR Methods). Note that the definition of the Oracle score is very similar to the Gaussian kernel reliability. We chose to keep the reported values instead of re-computing them, as the authors put a lot of effort into data curation and cleaning that would be difficult to reproduce.

**Figure 6:**
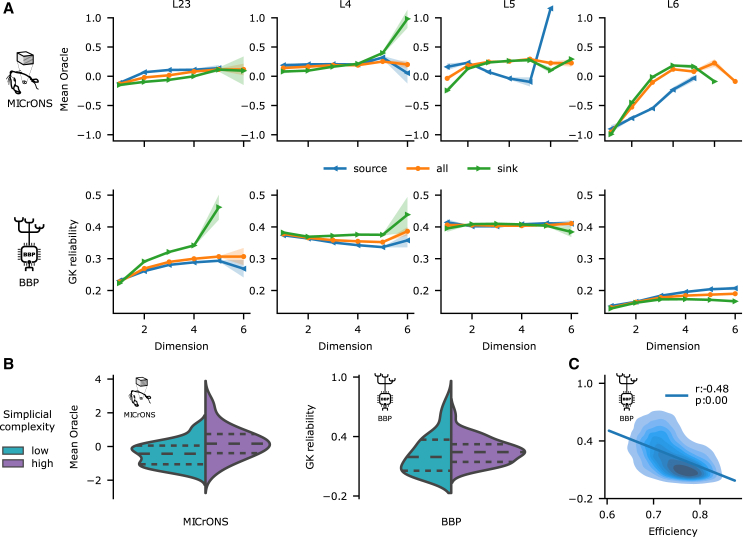
Reliability is shaped by the simplicial structure and network complexity (A) Reliability across simplices split by layer for both the MICrONS (Oracle score, top) and BBP (GK reliability, bottom) connectomes. Dots denote mean values and shaded regions are the standard error of the mean. The MICrONS reliability values have been *Z* scored in order to pool all sessions together maximizing the density of the activity recordings. The roles of the source and target neurons are flipped from the information receiving layers (2–4) to the readout layers (5–6). (B) The centers of the 100 most complex neighborhoods have a mean reliability that is significantly lower than the centers of the 100 least complex ones; with *p* values of $3.68\times 10^{-7}$ and $1.56\times 10^{-3}$ on the Kruskal-Wallis test for MICrONS and BBP, respectively. (C) The efficiency of a neighborhood is anti-correlated to the neuronal reliability of its center.

On the single neuron level, we considered reliabilities separately for neurons in individual layers. We found that reliability increased with cortical depth (moderately for the BBP data, slightly for MICrONS) until layer 5, after which it dramatically dropped in layer 6. Furthermore, for both datasets, the reliability of a neuron increased with the maximal simplex dimension it participates in (Figure 6A, orange curves), although this increase was weaker when the baseline reliability in a layer was already high, indicating that reliability cannot be raised over a certain ceiling value. Moreover, in layers 5 and 6, typically associated with cortical-cortical and cortical-thalamic outputs, respectively, we found that sources of simplices are more reliable than sinks (see STAR Methods, Figure 6A blue vs. green curves), while the opposite is true for the other layers. These trends were clear in the BBP dataset but were inconclusive in MICrONS. This could be related to the increasing sparsity of the calcium imaging data across layers in MICrONS. Indeed, when pooled across sessions, while up to 35% of the neurons are co-registered in layers 2/3, this drops to 15% and 3% in layers 5 and 6. Within a single scan, the data were too sparse to make a layer by layer split; the general trend of reliability increasing with simplex dimension was still observed, however (Figure S8).

On the level of neighborhoods, we contrasted the reliability of individual neurons with their neighborhood complexity for both datasets. Although we found a negligible correlation over the entire connectomes (Figure S14), we found that between the *champions*, i.e., the 100 neighborhoods that either minimize or maximize simplicial complexity, there is a statistically significant difference in their reliability (Figure 6B). Finally, the efficiency of a neighborhood is anti-correlated to the reliability of its center (Figure 6C).

In summary, network complexity increases reliability locally in two ways: On the node level, reliability increases with simplex dimension; on the subnetwork level, the most complex neighborhoods have significantly more reliable centers than their least complex counterparts. However, the strongest effect of network complexity is to increase the reliability of the network as a whole. However, this comes at the cost of lowered efficiency in reliability-promoting neighborhoods. The picture that emerges is one of low-complexity neighborhoods encoding information highly efficiently, but also robustly due to their interconnections with high-complexity neighborhoods that increase the reliability of the network as a whole.

## Discussion

We have investigated the role of the immense diversity of the structure found in neuronal networks of different species at cellular resolution. While the presence of wide, long-tailed degree distributions was already known, we found similarly long-tailed distributions of a variety of network metrics, in particular those capturing participation in the higher-order structure of the network. This persisted even in the face of normalization with respect to strong control models. Further, we found that participation of neurons in the higher-order structure was correlated with previously characterized overexpression of bidirectional connectivity.^45^^,^^61^ This gives rise to subnetworks centered on individual neurons (i.e., neighborhoods) that differ greatly along a spectrum of topological complexity. We linked this spectrum of complexity to circuit function, demonstrating that less complex subnetworks encode information with little redundancy and hence, efficiently. Going beyond abstract theory, we confirmed the efficiency of the subpopulations in a simulated stimulus classification experiment.

To investigate the role of more complex subnetworks, we ran simulations where we increased or decreased the degree of network complexity. The need to study the structure-function relation with connectome manipulations has been pointed out before in the study by Reid et al.^69^ We found that manipulations changed the mean reliability significantly on a global (whole-network) level, increasing as complexity increased. Additionally, for a given rewiring algorithm, the amount of change is dependent on the fraction of connections rewired. The strength of the effect was stronger for algorithms that decreased complexity, demonstrating that it is easier to destroy than to build the intricate wiring characterizing cortical circuits. In summary, more complex subnetworks promote the robustness of the code and play a supporting role by globally increasing the reliability of the neuron responses in evoked activity.

The contrast between reliability-enhancing and efficiently coding neurons is related to the previously characterized contrast between “soloists” and “choristers.”^61^^,^^62^ We indeed confirmed their results relating connectivity and population coupling and extended them to demonstrate the importance of higher-order structures. The importance of local connectivity for promoting reliable responses has also been observed before,^30^ but we were able to characterize connectivity motifs that promote reliability. Similarly, functional cell assemblies, which provide robust coding through redundancy, have been demonstrated to emerge through fewer synaptic connections in networks with complex higher-order structure.^32^ This is crucial for BNNs, because unlike artificial neural networks, they are tightly constrained in terms of their connection count: the immensely dense packing of neuronal wiring shows that a given function must be implemented with the least number of synapses possible.^70^ In summary, we confirm, extend, and link several known structural and functional features of biological neural networks into a single, coherent, and quantitative theory. The crucial aspect linking these features is consideration of the higher-order structure of the network, i.e., going beyond pairwise interactions.

Note that there are other notions of efficient coding, two of which are extensive in the literature. First, coming from information theory, the “efficient coding hypothesis,” proposed originally by Attneave^17^ and Barlow,^18^ links the statistics of natural stimuli to those of the neuronal response. Since natural stimuli are sparse, this theory proposes that an efficient code must also be sparse in the sense that it minimizes the number of active neurons, while preserving the amount of information (in an information theoretical sense). Second, a notion coming from dynamical systems, which posits that information is stored in a network in the form of disjoint basins of attraction in the space of network states, called patterns.^19^^,^^20^^,^^21^ The “network’s capacity” is the maximal number of patterns it can store, and a network is efficient when it has or it is close to maximal capacity. While these two notions of efficient coding have vastly different theoretical foundations, they also exhibit an efficiency-robustness tradeoff: the first, between minimizing the number of active neurons while still remaining “overcomplete” (i.e., containing enough features to encode any stimulus arbitrarily well) and the second, between the number of patterns that can be stored and the size of the basins of attraction, which determines robustness against noise.

Interestingly, theoreticians have found a class of networks that are close to maximal capacity, where reciprocal connections and higher-order motifs are overexpressed.^46^ However, in contrast to BNNs, they can explain this overexpression with the wide distributions of out-degrees; we speculate that BNNs are a specific subclass of such networks. Furthermore, Iatropoulos et al.^71^ explored a plasticity rule that explicitly improves robustness in simplified networks and contrasts it with one that maximizes network capacity. They found that the first rule leads to higher density of connections, while the latter leads to lower density. This is consistent with our results, given that higher density networks also have higher simplex counts. We extend the finding beyond connection density to simplex counts, which can be increased to some degree independently of connection density. Additionally, we show that both versions, i.e., high and dense subnetworks, can and do coexist in BNNs, as they are characterized by complex and heterogeneous connectivity. In summary, we also confirm, extend, and relate several predictions and theories for simplified neural networks.

On the other hand, what makes a network computationally powerful has also been explored through the lens of complex dynamical systems. It has been observed that maximal computational capacity arises at “the edge of chaos,”^22^^,^^23^ that is, at the boundary where the system transitions from ordered to chaotic dynamics. Moreover, it has been observed that the brain might be able to tune itself and evolve toward this region in what is known as self-organized criticality (SOC)^25^; though it remains unclear whether operating at the edge of chaos drives high performance or is a byproduct of the brain’s need to remain functional.^72^ Nonetheless, it has been shown that the brain’s architecture influences the shape of the criticality boundary. Legenstein and Maass^23^ found that in biologically detailed microcircuits with distance-dependent connectivity, the critical regions are determined by the connectivity strength and the level of heterogeneity of connection probability. However, Tadić and Melnik^24^ and Kilic and Taylor^73^ found that not only the pairwise connectivity structure but also the higher-order structure of the network or its activity are a driving force for the system achieving SOC. In the study by Markram et al.,^50^ it was shown that the BBP network we used in this work is at the boundary of criticality. It was furthermore confirmed for this network that the presence of higher-order structure affects the transition from asynchronous to synchronous activity^30^ and that neuronal reliability increases toward the transition boundary.^74^ This provides a link between the concepts explored in this work and the work on SOC. Interestingly, when we increased by an 8-fold the number of reciprocal connections along simplices, the system had a phase transition from asynchronous to synchronous activity, but it did not do so in the random control (Figures S13B2, S13D1, and S13D2). This further shows that indeed critical regions depend on the higher-order structure and not only on the pairwise interactions. Our findings suggest new possibilities for future research into the connection between attaining SOC and neighborhood simplicial complexity.

Beyond the general ideas outlined earlier, we also revealed more specific results relating to the roles of cortical layers that align with their known roles as inputs or outputs. Our notion of higher-order interactions is based on the presence of simplices, which are motifs with strong directionality, i.e., they have an input or *source* side and an output or *sink* side. In the simulation data, we found that, for neurons in layers 2, 3, and 4, efficiency decreased and reliability increased from the source to the sink of a simplex, while in layer 6, this was reversed. Neurons in layer 5 behaved as layer 6 with respect to efficiency, and the picture was non-conclusive for reliability, although they were overall the most reliable population.

The *in vivo* data were more sparsely sampled than the simulated data, leading to noisier results. In fact, the data were too sparse to estimate efficiency or to analyze reliability separately for individual layers within a given recording session. However, the per-layer analysis of reliability could be performed when pooling data across sessions, though the results were not conclusive, as the number of sample neurons decreased toward the deeper layers. Layer 5 is associated with extensive intratelencephalic, i.e., cortico-cortical outputs and layer 6 with cortico-thalamic outputs.^75^^,^^76^ Therefore, our interpretation is as follows: after inputs into a circuit arrive canonically in layer 4, local connectivity structure is optimized to enhance reliability while progressing activity forward. Within output layers, simplicial structure still increases reliability and enhances efficiency specifically of the output neuron population.

This view is compatible with the theory of “reservoir computing” or “liquid state machines,”^77^ which, in the context of neural circuits, posits that circuit inputs are first projected into a high-dimensional activity state and from there projected to a lower-dimensional readout. Seen in this context, we characterized connectivity mechanisms that increase the robustness of the higher dimensional projection (ironically reducing the dimensionality of that state) and increase the amount of information contained in the readout. In summary, our hypothesis predicts increasing robustness as the goal for the internal representation of a local circuit and increasing efficiency as the goal for its output, with specific structures of connectivity associated with both goals and embedded into a single network that promotes the reliability of the whole system. Our work can therefore be used in the future to link also this theory to the topics of non-random connectivity, efficiency, and soloist/chorister spiking dynamics.

More specific neuronal roles could be studied when considering larger volumes or full brain connectomes with co-registered activity. Although these data are not yet available at the micro-scale, there is promising potential for it to become accessible for small animals in the future. On the other hand, human connectomes at the meso-scale have been studied with topological methods before^33^^,^^34^^,^^36^ and have been found to have even more complex architectures, e.g., human brain connectomes have been found to have undirected simplices of up to 16 and 20 dimensions, in networks of only 1,115 nodes representing different brain regions.^34^ Furthermore, their higher-order structure has been found to have functional relevance.^33^^,^^78^^,^^79^^,^^80^ Interestingly, Tadić et al.^34^ does not only count undirected simplices but also how they are embedded in the full network via what is known as *q*-analysis. In short, in each dimension, one can divide all undirected simplices of dimension *q* into classes, where all the simplices in one class are those where one may travel from one simplex to another by passing through lower dimensional ones. The number of classes in each dimension is called the *number of q-connected components* and is a way of succinctly describing the higher-order structure of the network. The theory of *q*-analysis was extended to directed networks in the study by Riihimäki.^81^ This opens a compelling avenue for future research, using *q*-analysis of the level of neighborhoods and its impact on the efficiency-robustness tradeoff. Notably, neighborhoods with high simplicial complexity maximize the number and dimension of simplices in a highly nested and difficult-to-quantify manner. We conjecture, however, that once normalized by size, these neighborhoods minimize the number of *q*-components in each dimension, providing a means to quantify their intricate nested structures.

Our view sees trial-to-trial variability as a problem that needs to be overcome for reliable cortical function. This appears to be in contrast to existing theories where variability explicitly encodes uncertainty about a stimulus.^82^^,^^83^ However, we believe the views are compatible. First, the theoretical work sees uncertainty encoded primarily on the population level,^83^ while we measured reliability on the single-cell level. Second, we only investigated the prevalence of variability, not its structure and how it relates to stimuli. Finally, we found remaining uncertainty in the responses of even the most reliable cells. Furthermore, our results demonstrate a wide spectrum of reliability values and that the mechanisms to reduce it are not uniformly deployed. It is therefore possible that they are deployed in ways that implement the theories of explicit encoding of uncertainty. Moreover, we do not study the effect of correlations between neurons of their trial-to-trial variability, i.e., of noise correlation, on the network’s efficiency. A common theme on this body of work is that noise correlations are not independent of the network structure.^84^ Investigating how the architecture of BNNs shapes uncertainty encoding and the correlational structure of noise would constitute interesting follow-up work.

Many of our conclusions were drawn from the analysis of electron microscopic connectomes, primarily the MICrONS data,^49^ which while reconstructed with boundary-pushing methods is still imperfect. However, we have demonstrated that our network metrics are robust against reconstruction mistakes at the reported error rates. Our conclusions relating to efficiency of coding are based on simulations of a morphologically detailed model of cortical connectivity.^50^ They could be confirmed in the future when more or more densely sampled electron microscopic connectomes with co-registered activity data become available. In the meantime, the qualitative agreement between MICrONS and BBP data in most characterized trends indicates the validity of our model predictions. Quantitatively, non-random trends were slightly weaker in the BBP model than in the MICrONS data, which indicates that the effects observed in the *in silico* model might be even stronger in biology.

While in biology synaptic connections are associated with weights that are lognormally distributed, we considered exclusively unweighted network metrics. A standard procedure in topological data analysis to turn an unweighted metric into a weighted one is to compute the unweighted version repeatedly while filtering the network with a sliding weight threshold. There is a promising future avenue of research, extending our metrics to weighted metrics, enabling investigators to disentangle the effect of the low versus high strength of synaptic connections. However, such an analysis would require measuring the synaptic strength of millions of connections, which is at the moment not biologically feasible.

Experimental research has demonstrated several ways in which neuronal connectivity is non-random with significant higher-order structure.^33^^,^^35^^,^^37^^,^^41^^,^^51^ Moreover, small models with simple dynamics have shown the relevance of higher-order motifs in the observed activity^85^^,^^86^^,^^87^ while *in silico* research has shown that the complexity is largely driven by neuronal morphologies.^88^^,^^89^^,^^90^ At the same time, in the field of computational neuroscience, the bulk of research is conducted in models with simple connectivity based mostly on pairwise statistics that do not capture the entire complexity of the structure. This is in part due to the difficulty of building such complex networks as well as the need for a framework to quantitatively and systematically describe the higher-order structure. Both are required to demonstrate how this structure matters for the function of a neural circuit, if at all. Our work provides a theoretical framework, based on counting simplex motifs, that links and unifies the diverse aspects of non-random higher-order structure that have been found before. In contrast to global metrics of network complexity (e.g., small-world coefficient or assortativity), simplex counts also provide local metrics of node and edge centrality. These can be used to link functional properties of the neurons to their location in the network (Figures 4A and 4B). Moreover, possibly due to their feedforward nature, simplices have been shown to be functionally meaningful.^31^^,^^32^^,^^59^^,^^91^

Other motifs have been studied or counted before. Song et al.^92^ and Brunel^46^ count all motifs on 3 and up to 5 nodes, respectively. Parmelee et al.^86^ and Curto et al.^87^ define other types of functionally meaningful motifs, which they call “core motifs” and “robust motifs,” respectively. In contrast to these, counting simplices can be done more systematically and has been implemented efficiently.^93^ On the other hand, counting all motifs on *n* nodes present in a network of *N* nodes can only be done for small *n* due to a combinatorial explosion. Indeed, there are $\left(\begin{array}{l} N\\ n \end{array}\right)$ subnetworks on *n* nodes that need to be classified into $2^{2\left(\begin{array}{l} n\\ 2 \end{array}\right)}$ possible network types and further classified into motifs. Similarly, even listing core or robust motifs concretely above dimension 5/6 remains an open problem. Finally, *n*-cycles (i.e., cyclic motifs on *n*-nodes) have similar functional and generalization properties across dimensions as *n*-simplices. However, while counting both have the same worst-case complexity,^94^ in practice, simplex counts can be computed more efficiently, particularly in large, sparse networks.

Overall, our results and the cited previous research demonstrate that the complex architecture of BNNs provides a substrate to regulate the efficiency-robustness tradeoff through the existence of interconnected low- and high-density subpopulations within its heterogeneous connectivity.

### Limitations of the study

While simplex counts have been efficiently implemented, they can still be computationally prohibitive for certain networks. Heuristically, the computation is harder for larger, denser networks or those that have long fat tail degree distributions. It is also slowed significantly by high-degree nodes with high-degree neighbors; thus, computation time is highly related to the existence of a rich club in the network. However, the complexity of the computation cannot be clearly estimated *a priori*, since one cannot predict the higher-order structure just from pairwise metrics. For example, while we were able to count simplices in a network of 4.2 million nodes and 2 billion edges modeling the full somatosensory cortex of a rat’s brain,^90^ counting the number of simplices on an electron microscopy reconstruction of a central region of the adult *Drosophila* brain, a network of 25,288 nodes and 3.7 million edges,^95^ remains prohibitive due to the highly nested structure of its subnetworks. In the advent of larger and larger datasets becoming available, future work should address how to modify these methods or simplify the connectivity networks in order to address this limitation. These modifications could be motivated from the functional-structural implications found in this work in the micro-scale.

## Resource availability

### Lead contact

Requests for further information and resources should be directed to and will be fulfilled by the lead contact, Daniela Egas Santander (daniela.egassantander@epfl.ch or daniela.egas.math@gmail.com).

### Materials availability

This computational study did not generate new unique materials.

### Data and code availability

• Reliability and structure data have been deposited at Zenodo and are publicly available as of the date of publication at https://doi.org/10.5281/zenodo.10812496.
• This paper analyzes existing, publicly available data, accessible at DOIs listed in the key resources table.
• All original code has been deposited at GitHub and is publicly available as of the date of publication. DOIs are listed in the key resources table.
• Any additional information required to reanalyze the data reported in this paper is available from the lead contact upon request.

## Acknowledgments

The authors would like to thank Guillaume Tauzin for his help with Poetry-related questions in relation to connalysis, Elvis Boci for his help with visualizations, and Steeve Laquitaine for comments on an earlier version of this manuscript. This study was supported by funding to the Blue Brain Project, a research center of the École polytechnique fédérale de Lausanne (EPFL), from the Swiss government’s ETH Board of the Swiss Federal Institutes of Technology.

## Author contributions

Conceptualization, D.E.S., C.P., J.L., J.P.S., and M.W.R.; data curation, D.E.S., C.P., A.E., J.L., J.P.S., and M.W.R.; formal analysis, D.E.S., C.P., A.E., J.L., J.P.S., and M.W.R.; investigation, D.E.S., C.P., J.L., and J.P.S.; methodology, D.E.S., C.P., A.E., J.L., M.S., J.P.S., K.H., R.L., and M.W.R.; project administration, M.W.R.; software, D.E.S., C.P., A.E., J.L., M.S., J.P.S., and M.W.R.; supervision, M.W.R.; validation, D.E.S., C.P., A.E., J.L., J.P.S., and M.W.R.; visualization, D.E.S., C.P., and A.E.; writing – original draft, D.E.S., C.P., A.E., and M.W.R.; writing – review and editing, D.E.S., C.P., A.E., J.L., M.S., J.P.S., K.H., R.L., and M.W.R.

## Declaration of interests

J.L. currently also holds a position at Printful. This affiliation was established after the majority of his contributions to this manuscript and is unrelated to the work presented here. R.L. also serves as the Director of MiiST-UK, Mathematical Innovation in Science and Technology, Edinburgh, Scotland, UK. This role is unrelated to this manuscript. Finally, some authors hold minimally relevant patents related to this work, which are listed below.

• K.H.: US11569978B2 and US11652603B2.
• K.H. and R.L.: US11893471B2, US11972343B2, US20240176985A1, US20230297808A1, US20230351196A1, US11580401B2, US11663478B2, US20190378000A1, and US20190378007A1.
• K.H., R.L., and M.W.R.: US11615.

## STAR★Methods

### Key resources table

**Table:** 

REAGENT or RESOURCE	SOURCE	IDENTIFIER
**Deposited data**		

C. elegans connectome	Witvliet et al.^47^	https://wormwiring.org/pages/witvliet.html; https://www.wormatlas.org/images/NeuronType.xls
Drosophila larva connectome	Winding et al.^48^	https://www.science.org/doi/suppl/10.1126/science.add9330/suppl_file/science.add9330_data_s1_to_s4.zip
MICrONS connectome	MICrONS Consortium et al.^49^	https://doi.org/10.5281/zenodo.8364070
BBP connectome	Markram et al.^50^	https://doi.org/10.5281/zenodo.10599103
Reliability & structure dataset	This paper	https://doi.org/10.5281/zenodo.10812496

**Software and algorithms**		

Reliability & structure	This paper	https://github.com/BlueBrain/reliability-and-structure
Connectome-Analysis	This paper	https://github.com/BlueBrain/connectome-analysis
Connectome-Utilities	Reimann et al.^96^	https://github.com/BlueBrain/ConnectomeUtilities
Connectome-Manipulator	Pokorny et al.^67^	https://github.com/BlueBrain/connectome-manipulator
TriDy/TopoSampling pipeline	Conceição et al.^64^; Reimann et al.^63^	https://github.com/JasonPSmith/TriDy; https://github.com/BlueBrain/topological_sampling/tree/merge_samples
TriDy-tools	This paper	https://github.com/jlazovskis/TriDy-tools
assemblyfire	Ecker et al.^32^	https://github.com/BlueBrain/assemblyfire

### Method details

#### Structural datasets

We analyzed the connectivity in the following four openly available data sets recorded at cellular resolution. We used the ConnectomeUtilities Python package^96^ to turn these data sets into the common “ConnectivityMatrix” representation format for further analysis.

**C. elegans**^47^ Electron microscopy reconstruction of the full brain connectivity of the adult C. elegans (Dataset 8; last stage). The data set consists of 324 neurons and 2186 connections formed by 7970 chemical synapses. We did not include electrical synapses in our analyses. Additionally, we discarded 105 isolated neurons of the connectome, i.e., neurons without incoming or outgoing connections.

**Drosophila larva**^48^ Electron microscopy reconstruction of the full brain connectivity of the Drosophila larva. We included both hemispheres but only considered “axo-dendritic” connections. The resulting data set consists of 2956 neurons and 63,545 connections formed by 234,958 synapses.

**MICrONS**^49^ Electron microscopy reconstruction of a cubic millimeter volume spanning all 6 layers of mouse primary visual cortex and three higher visual areas. A reformatted version for easy use with ConnectomeUtilities is available on Zenodo.^97^ We excluded the boundaries and restricted the data set to excitatory types. The resulting connectome consists of 9619 neurons and 520,477 connections formed by 610,261 synapses.

**BBP**^50^ Morphologically detailed model of the rat somatosensory cortex,^50^ consisting of more than 210k biophysically detailed neurons belonging to 55 different morphological and 207 different morpho-electrical types. The neurons are connected by over 400M synapses with probabilistic transmitter release and six distinct forms of short-term dynamics of depression and facilitation. The whole circuit can be divided into seven hexagonal columns. To avoid any edge effects, we restricted all analyses (connectivity and activity) to neurons within the central hexagonal column. This data set is available on Zenodo^98^ and can be loaded with ConnectomeUtilities. Within that column, we only considered the sub-circuit of 6,717,001 connections between 26,567 excitatory neurons. In the context of connectome manipulations (see Section connectome manipulation pipeline), we refer to this data set as *baseline connectome*.

#### Activity dataset for MICrONS

We analyzed functional data from the MICrONS dataset.^49^ The open source dataset contains calcium traces and deconvolved spike trains of a sparse set of neurons, which are co-registered to the structural dataset. We analyzed data from eight recording sessions from the v661 version of the “minnie65_public” release. The sessions were selected based on two criteria: at least 1000 neurons were scanned and at least 85% of them were co-registered to the structural data. Neurons with a non-unique identifier (2.8% neurons/session on average) were filtered out. Averaged across sessions, 2% of the neurons in the connectome (243 neurons/session) had activity data co-registered to them. Moreover, as calcium imaging is biased towards the superficial layers, the layer-wise distribution of co-registered cells is not uniform, but as follows: layer 23: 36.5%, layer 4: 44.9%, layer 5: 15.3%, and layer 6: 3.3%. Spike trains of neurons restricted to the structural connectome outlined above (243 neurons/session on average, 1817 unique neurons in total, 2% and 15% of all cells respectively) were used to calculate coupling coefficients (see below). As the dataset already contained oracle scores (see below) we did not calculate reliability from the raw spike trains.

#### Activity dataset for BBP

We ran circuit simulations of the morphologically detailed model of the rat somatosensory cortex,^50^ which was converted to the SONATA format^68^ in order to create manipulated versions of its connectome (see Section connectome manipulation pipeline). We simulated the entire circuit (original and manipulated versions) using the CoreNEURON simulator^99^ and recorded the spiking activity of the network in response to a series of stimuli applied to the thalamocortical input fibers. As in Reimann et al.,^63^ the 2170 input fibers were partitioned into 100 clusters of adjacent fibers. Ten such clusters were randomly drawn to form a spatial stimulus pattern. For each cluster belonging to such a pattern, a spike train with a firing rate of 75 Hz was independently generated by an adapting Markov process (with 100 ms adaptation time constant) for 75 ms, followed by a 125 ms blank period with 0.5 Hz firing. We used a random sequence of eight such spatial patterns in two simulation protocols, results of which are available on Zenodo^100^:

**Reliability protocol** We applied a total number of 80 stimuli (i.e., 10 repetitions per pattern) during 16 s. We reran the experiment 10 times with different simulator seeds using the exact same (random) sequence of stimulus patterns and input spike trains.

**Classification protocol** We applied a total number of 800 stimuli (i.e., 100 repetitions per pattern) during 160 s. We used a single simulator seed without repeating the ex periment.

#### Random networks

This section contains definitions of the different random connectivity control models and how these were generated. A summary of these is listed in Table 1.

The first three controls in Table 1 modify the global structure of the network whilst keeping one property fixed. They are listed in order of complexity. The *Erdős–Rényi* controls (ER) are random directed networks, where each edge is added independently at random with a fixed probability *p* given by $p=\frac{E}{N\left(N-1\right)}$, where *E* and *N* are the number of edges and nodes in the original network, thus keeping the global connection probability fixed.

The *directed configuration model* controls (CM) are random networks with (approximately) fixed in- and out-degree sequences, i.e., the vectors whose entries are the in- and out-degrees of all the nodes in the network. To construct such controls we wrote the original network in coordinate format i.e., we wrote its edges as two vectors sources and targets, such that each pair (sources[i],targets[i]) was a directed edge in the original network. We then shuffled the entries of both vectors independently, which gave a new directed network, with the same degree sequence as the original. Note that this new directed network might contain loops (edges of the type (a,a)) or parallel edges (multiple edges with the same source and target). We call these degenerate edges and we removed them. The effect of this removal is that this construction only approximated the original degree sequence. However, the density of degenerate edges tends to decrease as the number of nodes increases,^101^ so the approximation was close. Indeed, while on average ∼5% of the edges were lost for the CM controls of C. elegans, only 1.08%,1.15% and 0.875% of the edges were lost for Drosophila, MICrONS and the BBP data sets respectively.

The *distance dependent model* controls (Dist) are random networks where the edges are added independently at random with a probability that is exponentially decreasing with distance. More precisely, for any pair of neurons, let *d* be the Euclidean distance between the position of their cell bodies. Then, their probability of connection is given by

$$p\left(d\right)=\alpha \cdot e^{-\beta \cdot d}\text{,}$$

where α (the probability at distance zero) and β (the decay constant) were determined from the connectivity of the original graph. In order to do so, we computed the pairwise distance between all pairs of nodes in the network and binned them into groups according to this distance. The connection probabilities were estimated in each bin by dividing the number of existing edges by the number of total pairs in that bin. The model coefficients were then determined by fitting the exponential probability function p(d) to these data points. We did this only for the MICrONS and BBP data sets, obtaining $\alpha \approx 0.067,\beta \approx 1.18\times 10^{-2}\mu m^{-1}$ and $\alpha \approx 0.111,\beta \approx 7.05\times 10^{-3}\mu m^{-1}$ respectively. For C. elegans and Drosophila the Euclidean distances between cell bodies might not be the most relevant factor determining connection probability.

The last two controls in Table 1 are used to control for the possible location of reciprocal connections, while keeping the number of simplex counts close to those of the original graph (specially for large sparse graphs). The *Underlying* controls (Und) are random networks with the same underlying undirected graph as the original and the same number of reciprocal connections. To generate such controls, we first extracted the underlying undirected graph of the original network i.e., the undirected graph with the same nodes as the original and an undirected edge between any two pair of nodes that are connected by a reciprocal or unidirectional edge. We then chose an ordering of the nodes and oriented all edges of the undirected network to go from a smaller to a larger node. Finally, we added the same number of reciprocal connections as in the original graph at random.

The last control, the *rc-Shuffled* control (rcSh) is the tightest control of all. These are random networks where all of the structure of the original network is kept, except the location of the reciprocal connections. To build such a control, for every reciprocal edge in the original network we chose one direction to delete at random. Then we introduced back at random the same number of reciprocal connections along the existing edges.

#### Network metrics

In a network *G* the *in-degree* and *out-degree* of a node *v* is the number of incoming and outgoing connections from *v*, respectively, and the *total degree* of *v* is the sum of both. We call the *reciprocal degree* of *v* the number of reciprocal connections on *v*, i.e., the number of nodes in *G* that that map to and from *v*.

An *n-simplex* in *G* is a set of n+1 nodes which are all to all connected in feedforward fashion. That is, there is a numbering of the nodes 0,1,…n such that whenever i<j there is an edge from *i* to *j* in the network *G*. We call *n* the *dimension* of the simplex, the numbering 0,1,…n the *position* of the nodes in the simplex and the edges

0→1→2→…→n−1→n

the *spine* of the the simplex. A simplex is *maximal* if its nodes and edges are not contained in a higher dimensional simplex.

We weight the edges of *G* with the maximal dimension amongst all the simplices it participates in and we denote by $G_{k}$ the subgraph of *G* on the edges with weight ≥k. That is, the edges in $G_{k}$ belong to at least one simplex of dimension *k*. In particular $G_{1}=G$, since every edge is a 1-simplex. We then compute the *reciprocity of G at dimension k*, denoted ${\text{rc}}_{k}$, as the percentage of reciprocal edges of $G_{k}$

$${\text{rc}}_{k}=\frac{\text{number}\;\text{of}\;\text{reciprocal}\;\text{edges}\;\text{of}\;G_{k}}{\text{number}\;\text{of}\;\text{edges}\;\text{of}\;G_{k}}\times 100\text{.}$$

We plot the curves $k\mapsto {\text{rc}}_{k}$ for all connectomes and their corresponding controls in Figure 2D.

The simplicial structure of *G* can be used to compute (weighted) averages of any node based property as follows. Let *N* be the number of nodes of *G* and $\vec{v}\in R^{N}$ be a vector given by a node based property, e.g., node degree or reliability. For any dimension *k* and any possible position 0≤pos≤k within a *k*-simplex, we denote by $\text{mean}\left(\vec{v},k,pos\right)$ the mean of the values of $\vec{v}$ of the nodes in position pos of *k*-simplices, counted with multiplicity. More precisely, if $s^{1},\ldots ,s^{t}$ is a list of all *k*-simplices, let $\vec{v}\left(s_{pos}^{i}\right)$ be the value of $\vec{v}$ of the node in position pos of $s^{i}$, then we define $\text{mean}\left(\vec{v},k,pos\right)$ as the mean

$$\text{mean}\left(\vec{v},k,pos\right)=\text{mean}\left\{\vec{v}\left(s_{pos}^{1}\right),\vec{v}\left(s_{pos}^{2}\right),\ldots ,\left(s_{pos}^{t}\right)\right\}\text{.}$$

We denote by $\text{mean}\left(\vec{v},k,all\right)$ the weighted mean of $\vec{v}$ on the nodes that are in *k*-simplices (counted with multiplicity) regardless of position, i.e.,

$$\text{mean}\left(\vec{v},k,all\right)=\text{mean}\left\{\vec{v}\left(s_{pos}^{1}\right),\vec{v}\left(s_{pos}^{2}\right),\ldots ,\left(s_{pos}^{t}\right)|0\leq pos\leq k\right\}\text{.}$$

We plot the curves $k\mapsto \text{mean}\left(\vec{v},k,all\right)$ in Figures 3B, 4A, 4C, and 6A, for different structural and activity properties of the nodes. In the last three cases, we further plot $k\mapsto \text{mean}\left(\vec{v},k,pos\right)$ for pos=0 or pos=k i.e., the source respectively target neurons of a simplex.

#### Neighborhood complexity

Let *G* be a network and *v* be a node in *G*. We call the *neighborhood of v* the subgraph of *G* on all the nodes in *G* that are connected to *v* and the edges between them. We denote this subgraph by $N_{v}$ and we call *v* its *center*. The *size of* $N_{v}$ is the number of nodes in it. In the absence of reciprocal connections, the size of $N_{v}$ is the total degree of *v* plus 1. However, in general we have that

$$\text{size}\left(N_{v}\right)=\text{total}\;\text{degree}\left(v\right)+1-\text{reciprocal}\;\text{degree}\left(v\right)\text{.}$$

The complexity of $N_{v}$ is determined by how similar or different it is from a random subgraph of a similar size. In order to quantify this, we generate a configuration model control of *G*. We denote this control $\hat{G}$ and by ${\hat{N}}_{v}$ the neighborhood of *v* in $\hat{G}$. Note that by construction $N_{v}$ and ${\hat{N}}_{v}$ are of similar size, particularly for large sparse graphs. Thus, we define the *neighborhood complexity of* $N_{v}$ as the distance between $N_{v}$ and ${\hat{N}}_{v}$. See Figure S5 for an explicit example. We measure this distance using simplex counts and define

$$\text{Simplicial}\;\text{neighborhood}\;\text{complexity}\left(N_{v}\right)=d\left(\bar{SC_{N_{v}}},\bar{SC_{{\hat{N}}_{v}}}\right)\text{,}$$

where $\bar{SC_{N_{v}}},\bar{SC_{{\hat{N}}_{v}}}$ denote the simplex counts of $N_{v}$ and ${\hat{N}}_{v}$ respectively linearly normalized to jointly lie between 0 and 1, and d denotes Euclidean distance. To test the robustness of our metric, we consider additionally a distance given by a metric that only takes into account pairwise interactions and define a degree based complexity metric as

$$\text{Degree}\;\text{neighborhood}\;\text{complexity}\left(N_{v}\right)=W\left(D_{N_{v}},D_{{\hat{N}}_{v}}\right)\text{,}$$

where $D_{N_{v}}$ and $D_{{\hat{N}}_{v}}$ are the total degree distributions of $N_{v}$ and ${\hat{N}}_{v}$ respectively and *W* is the Wasserstein distance between them, a classical distance between probability distributions.^102^ These two values are strongly correlated to each other (Figure S6D).

#### Reliability

We measured the reliability of spike trains to repeated presentations of natural movies in the MICrONS dataset, with the *Oracle score* reported by MICrONS.^49^ The score was computed as the jackknife mean of correlations between the leave-one-out mean across repeated stimuli with the remaining trial. The sparsity of the activity data set required us to pool Oracle scores across sessions for some analyses. Since the baseline Oracle scores have large variability across sessions (Figure S8A), we first z-scored the Oracle scores in each session and then average them across sessions.

To measure the spike timing reliability of a neuron in the BBP data set, we used the notion of *Gaussian kernel reliability*^66^ across repetitions of the same experiment. The repetitions were obtained from repeated simulations with different simulator seeds. Specifically, for a set of spike trains of a given neuron obtained from repetitions of the same experiment, we first convolved the spike trains with a Gaussian kernel with σ=10 ms. Since reliability is known to be confounded by the firing rate of a neuron,^103^ we removed this dependency by *mean-centering* the data (see Figure S15), i.e., by subtracting the mean of the convolved signal. Then, for each neuron we computed the cosine similarity between all pairs of repetitions of mean-centered signals and averaged the resulting values.

#### Connectome manipulation pipeline

We employed the recently developed Connectome-Manipulator Python framework,^67^ which allows rapid manipulations of the connectivity of circuits in the SONATA format,^68^ which we will denote by *sonata-connectomes*. The framework supports the creation of new sonata-connectomes (referred to as wiring), transplantation of connectivity features from other sonata-connectomes, and manipulations of existing sonata-connectomes (referred to as rewiring) on the level of connections and synapses. To create a manipulated sonata-connectome we require a connection probability model defining the connectivity together with parameter models specifying the physiological properties of the generated connectivity. We provide details for these in the following subsections. The adjacency matrices of all manipulated sonata-connectomes are available on Zenodo.^100^

#### Connection probability models

There were four categories of manipulations done on the baseline connectome: *remove reciprocity, increase reciprocity, reduce complexity, enhance complexity*. These are listed in Table 2 and we describe them explicitly here. All manipulations were restricted to connections between excitatory neurons in the central hexagonal column of the circuit.

In case of manipulations that *reduce complexity*, labeled OrderM, we globally rewired the connectome across the complexity spectrum based on five simplified stochastic connection probability models^67^^,^^104^ which had been fit against the baseline connectome beforehand, using the Connectome-Manipulator framework. The total numbers of connections in these simplified connectomes were closely matched to the number of connections in the baseline connectome (relative difference less than 0.1 %) using the iterative procedure described in Pokorny et al..^67^ Self-connections (autapses) were not allowed. We used the following simplified models:

**Order 1** Uniform connection probability between all pairs of pre- and post-synaptic neurons.

**Order 2** Distance-dependent connection probability; the probability depends on the Euclidean distance between pre- and post-synaptic neurons (conceptually equivalent to the *Distance model* of Table 1, but with the technical difference that it was modelled by the sum of a proximal and distal exponential function^67^). For fitting the model, we used a distance binning of 50 μm.

**Order 3** Bipolar distance-dependent connection probability; same as Order2 (including the binning parameters) but with a distinction between pre-synaptic neurons above or below post-synaptic neurons (in axial direction perpendicular to the cortical layers).

**Order 4** Offset-dependent connection probability; the probability depends on the axial and radial offsets between pre- and post-synaptic neurons (in a cylindrical coordinate system with axial direction perpendicular to the cortical layers). For fitting the model, we used an offset binning of 50 μm.

**Order 5** Position- and offset-dependent connection probability; same as Order4 but with additional dependence on the absolute axial position (i.e., cortical depth) of the pre-synaptic neuron. For fitting the model, we used an offset binning of 100 μm, and a position binning of 400 μm.

The other three categories of manipulations were based on adjacency matrices generated beforehand which we employed as deterministic connection probability models, i.e., containing only probability values of zeros and ones as determined by the matrix. We describe how these adjacency matrices where generated.

On the manipulations that *reduce reciprocity*, labeled RC−K, for selected reciprocal connections we removed one of the two directed edges at random. The reciprocal connections were selected from the spine of maximal *n*-simplices as follows (recall that the spine of an n-simplex is the set of edges 0 → 1 → 2 →… → n):

**RC – 1** Reciprocal connections on the spine of maximal *n*-simplices for n≥5.

**RC – 2** Reciprocal connections on the spine of maximal *n*-simplices for n≥5 and half (chosen at random) of the reciprocal connections that are on the spine of maximal *n*-simplices for n=4 that are not in the spine of a higher dimensional simplex.

**RC – 3** Reciprocal connections on the spine of maximal *n*-simplices for ≥4 .

**RC – 4** All reciprocal connections.

To control for the effect of the location of reciprocal connections we generated manipulations, denoted RC∗−K for 1≤K≤3. In each manipulation RC∗−K we removed the same number of reciprocal connections as in RC∗−K, but we did so at random rather than from maximal simplices of top dimension. Note in particular, that there is no RC∗−4, since it is not possible to remove all reciprocal connections at random.

On the manipulations that *increase reciprocity*, labeled RC+K, we added reciprocal connections to existing edges such that the percentage of reciprocal connections is approximately multiplied by a chosen factor $F_{K}$ across all maximal simplices. To do this, let *G* be the original connectome, let $G_{k}$ be the subgraph on edges that are on simplices of dimension *k* (see Subsection network metrics) and let $nrc_{k}$ be the number of reciprocal connections of $G_{k}$. For all dimensions k≥2, we selected at random $F*nrc_{k}$ non-reciprocal edges of $G_{k}$ that we turned into reciprocal connections. We did this for $F_{K}=2,3,4,5\;\text{and}=8$. To control for the effect of increased connectivity density we generated manipulations, denoted RC∗+K for all *K*, where we added the same number of connections as in RC+K at random in the excitatory subnetwork.

Finally, on the manipulations that *enhance complexity*, we use a custom algorithm presented by Reimann et al.^91^ which exchanges edges in order to maximize the number of simplices an edge participates in. Briefly, for each edge in the network, we computed its *maximal dimension of edge participation*, which is the maximum dimension of simplices it participates in. For each node in the network and we computed its *n-node participation* for all dimensions *n*, which is the number of *n*-simplices the node participates in. Then we removed *M* edges at random with maximal dimension edge participation smaller or equal to 3 (the middle dimension for this connectome) and added *M* new edges at random, where the probability of an edge a→b to be added was proportional to the participation of *a* and *b* in simplices above dimension 3. We performed these manipulations for M=100k,200k,300k,400k,500k,and670k and labelled these manipulations as *M*. Note that 670k is nearly 10% of all the connections.

#### Generating sonata-connectomes

When rewiring connectivity based on a given model of connection probability, existing connections may be deleted, but also new ones created which requires parameter models specifying the physiological properties of new connections. Following the pipeline of Pokorny et al.,^67^ we first fitted a “connection properties model” against the baseline sonata-connectome. This model captured parameter distributions from which physiological property values were then drawn during rewiring, such as: conductance (gamma), decay time (truncated normal), depression time (gamma), facilitation time (gamma), utilization of synaptic efficacy (truncated normal), and number of synapses per connection (discrete). All these parameter distributions were fitted in a pathway-specific way, i.e., separately for all 13×13 pairs of pre- and post-synaptic morphological types of excitatory neurons. Second, for assigning axonal delays, we fitted a “linear distance-dependent delay model” against the baseline sonata-connectome (with 50 μm distance binning) where the mean synaptic delay linearly depends on the Euclidean distance between the soma of a pre-synaptic neuron and the synapse location on the post-synaptic dendrite. Finally, in case rewired connections were to be established that already existed in the baseline sonata-connectome, there were two options: keeping them or re-generating them. For the manipulations that reduce complexity (OrderM), we re-generated all connections whereas for all other manipulations we kept existing connections as they were, i.e., we only deleted unused ones and generated new ones that did not exist before. In all cases, we used the option for reusing existing synapse positions, so that new synapses were placed on the post-synaptic dendrites at positions randomly sampled from existing synapses in the baseline sonata-connectome.

#### Coupling coefficient

Following the ideas of Okun et al.,^61^ we calculated coupling coefficient of a neuron *i* as the Pearson correlation of its activity with the activity of the rest of the circuit and denoted it $c_{i}$. More precisely,

$$c_{i}=\frac{\sum_{t}\left(f_{i}\left(t\right)-\mu_{i}\right)\times \left(f_{\neq i}\left(t\right)-\mu_{\neq i}\right)}{\sqrt{\sum_{t}{\left(f_{i}\left(t\right)-\mu_{i}\right)}^{2}\times {\left(f_{\neq i}\left(t\right)-\mu_{\neq i}\right)}^{2}}}\text{,}$$

where $f_{i}$ is either the neuron’s deconvolved Δ F/F for MICrONS, or its binned (with 5 ms bin size) spike train for BBP, $\mu_{i}$ is $f_{i}$’s mean, $f_{\neq i}\left(t\right)$ is the activity of the rest of the circuit i.e.,

$$f_{\neq i}\left(t\right)=\sum_{j\neq i}f_{i}\left(t\right)\text{,}$$

and $\mu_{\neq i}$ is its mean. Note that when the number of neurons is large, $f_{\neq i}\left(t\right)$ is approximately the summed activity of all neurons. Thus, we used this value instead for the BBP data for an efficient implementation.

Furthermore, $c_{i}$ values were normalized by z-scoring their differences from the average of 10 $c_{i}$ values from surrogate datasets. Control datasets were created by randomly shuffling spike times, i.e., keeping single cell firing rates. We choose this *per-cell* normalization as opposed to *global* normalization (in which one would z-score $c_{i}$ values by the mean and standard deviation of all neurons’ surrogate $c_{i}$ s) as the global one resulted in a multimodal distribution for the BBP data (Figure S16B1). The source of this artifact is that *in silico* we could record even the extremely low firing rate neurons, which lead to a low, secondary peak in the distribution (Figure S16B2). On the other hand, for the MICrONS dataset, both types of normalization returned virtually the same values (Figure S16A).

#### Efficiency of a neighborhood

We define the *efficiency of a neighborhood* as the dimension of its activity normalized by the size of its active subneighborhood i.e., the number of nodes in it that fire.^11^^,^^12^^,^^13^^,^^14^^,^^15^^,^^16^ We determined the dimensionality of the activity of the neighborhood using the correlation of the spike trains of its nodes. Thus, a neighborhood of maximum efficiency has maximally uncorrelated data and encodes more information, relative to its size.

Specifically, as for reliability, we first convolved the spike trains with a Gaussian kernel with σ=10 ms, to reduce the effect of noise and we call these the *spike signals*. A fraction of all the neurons (between ∼25% and 30%) remain silent during a simulation. For each node *v* in *G*, let l be the number of nodes in its neighborhood $N_{v}$ that fire and let ${\text{Corr}}_{v}$ denote the mean centered l×l matrix given by the pairwise Pearson correlation between the spike signals (see Figure 4B left for examples). We computed the singular values of ${\text{Corr}}_{v}$ via singular value decomposition (SVD) and we denote by $\vec{ss_{v}}\in R^{l}$ the cumulative vector of singular values normalized to lie between 0 and 1. The normalized dimension of the activity of $N_{v}$ is the fraction of the l nodes required to reach 0.9 of the cumulative spectrum i.e.,

$$Efficiency\left(N_{v}\right)=\frac{n_{0.9}}{l}\text{,}$$

where $n_{0.9}$ is the first position in $\vec{ss_{v}}$ at which 0.9 is reached (see Figure 4B right).

#### Classification

We used the neighborhood-based stimulus classification pipeline described by Conceição et al.^64^ and Reimann et al..^63^ We briefly summarize their pipeline here, which we refer to as the *single selection procedure* and refer the reader to the original papers for details on their method.

First, we computed the neighborhood metrics listed in Table S1 for the neighborhoods of all excitatory neurons within the excitatory subcircuit and called these the *selection parameters*. Second, amongst all excitatory neurons, we picked 50 neighborhood centers either: at random or those that maximize or minimize a given *selection parameter*. Third, we featurized the activity of the selected neighborhoods as a response to each stimulus and reduced it to a set of time series that we call the *feature vectors*. The featurization procedure was done in two generic ways. On one hand, following Reimann et al.,^63^ in what we call the *PCA method*, we applied principal component analysis to the binned spike trains of the union of the selected neighborhoods with a bin size of 20 ms. On the other hand, following Conceição et al.,^64^ we considered the *active subnetworks* of each neighborhood, which are the subgraphs of the neighborhood on the nodes which are active in a given time window; for each stimulus presentation we considered two time windows of 25 ms each. This reduced the activity to a set of time series of graphs (one per stimulus), which we then transformed into feature vectors by calculating their value of one of the network metrics listed in Table S1. Finally, we classified these feature vectors using a Support Vector Machine (SVM) and computed the accuracy and cross-validation error.

Note that this pipeline provides for each (selection, featurization) parameter pair a classification accuracy obtained only from the activity of 50 neighborhoods rather than the full circuit. Moreover, the sub-selection of the activity data is done purely based on the structural properties of the circuit, in particular its neighborhood structure. However, in most cases the featurization procedure uses both the structural and activity data except for the PCA method which is based in the activity values only. In particular, the number or percentage of non-zero features within the feature vectors (which are ultimately the information that enters the classifier) depend not only on the size of the union of the neighborhoods but also on the (selection, feature) pairing and (see Figure S11).

To test the effect of the neighborhood complexity on its efficiency as a classification readout we slightly modified the pipeline above and contrasted the results with the original. The modification was simple, and affected only the second step described above in which we replaced the single selection procedure by what we call the *double selection procedure*. Specifically, instead of selecting the neighborhoods amongst all excitatory cells, we first selected those on the top 1% of reciprocal density (high complexity) or the bottom 1% (low complexity). We then selected neighborhoods from these either at random or according to a given selection parameter and followed the same steps described above. All classification results are available on Zenodo.^100^

### Quantification and statistical analysis

The one sample t-test was used to test if simplex counts and number of reciprocal connections of a given connectome are significantly larger/lower from the expected values on their corresponding random network controls. To compare the means of activity metrics (e.g., reliability, coupling coefficient, efficiency) across groups the Kruskal-Wallis-Test was used instead, since these distributions were not normal. In both cases, the sizes of the samples and *p*-values are reported in the figure captions.

To evaluate the non-symmetry of the uni-modal reliability distributions for the different manipulated connectomes, we computed their skewness using the Fisher-Pearson coefficient.^105^ This parameter has a value of zero for normally distributed variables and a positive/negative value for unimodal distributions that are long tailed to the right/left.
