# Supplementary material for: Heterogeneous and higher-order cortical connectivity undergirds efficient, robust, and reliable neural codes
Source: iScience. 2024 Dec 12;28(1):111585. doi: 10.1016/j.isci.2024.111585 (PMC11751574; doi:10.1016/j.isci.2024.111585)
Supplement: Document S1. Figures S1–S16 and Table S1 [file mmc1.pdf]

iScience, Volume *28*

Supplemental information

Heterogeneous and higher-order cortical connectivity undergirds efficient, robust, and reliable neural codes

Daniela Egas Santander, Christoph Pokorny, András Ecker, Jānis Lazovskis, Matteo Santoro, Jason P. Smith, Kathryn Hess, Ran Levi, and Michael W. Reimann

Table S1: Feature and selection parameters, supplement to STAR Methods-Classification. Each parameter is marked by S, F or S+F, denoting that it is used for selection only, featurization only, or selection and featurization, respectively. We have split the parameters into three categories: *Activity*, which considers only the activity of the neighborhoods and not their network structure, *Topology*, which consider a topological metric of the active subgraphs and *Spectral*, which consider a spectral property of the active subgraphs. Detailed definitions of all network the metrics can be found in Conceição et al. [1].

Abbreviation	Parameter name	Use	Type
**pca**	PCA of activity	F	Activity
**ns**	Neighborhood size	S+F	Activity
**deg**	Neuron degree	S+F	Topology
**ideg**	Neuron in-degree	S+F	Topology
**odeg**	Neuron out-degree	S+F	Topology
**avdeg**	Average degree	S	Topology
**ec**	Euler characteristic	S+F	Topology
**nbc**	Normalized Betti coefficient	S+F	Topology
**fcc**	Clustering coefficient	S+F	Topology
**tcc**	Transitive clustering coefficient	S+F	Topology
***i*simplex**	i-simplex count	S	Topology
***i*contain**	i-simplex containment	S	Topology
**dc*i***	Simplex density	S+F	Topology
**asg**	Adjacency spectral gap	S+F	Spectral
**asl**	Adjacency spectral gap (low)	S+F	Spectral
**asr**	Adjacency spectral radius	S+F	Spectral
**blsg**	Bauer Laplacian spectral gap	S+F	Spectral
**blsl**	Bauer Laplacian spectral gap (low)	S+F	Spectral
**blsr**	Bauer Laplacian spectral radius	S+F	Spectral
**blsRg**	Reversed Bauer Laplacian spectral gaps	S+F	Spectral
**blsRl**	Reversed Bauer Laplacian spectral gap (low)	S+F	Spectral
**blsRr**	Reversed Bauer Laplacian spectral radius	S+F	Spectral
**clsg**	Chung Laplacian spectral gap	S+F	Spectral
**clsh**	Chung Laplacian spectral gap (high)	S+F	Spectral
**clsr**	Chung Laplacian spectral radius	S+F	Spectral
**tpsg**	Transition probability spectral gap	S+F	Spectral
**tpsl**	Transition probability spectral gap (low)	S+F	Spectral
**tpsr**	Transition probability spectral radius	S+F	Spectral
**tpsRg**	Reversed transition probability spectral gap	S+F	Spectral
**tpsRl**	Reversed transition probability spectral gap (low)	S+F	Spectral
**tpsRr**	Reversed transition probability spectral radius	S+F	Spectral

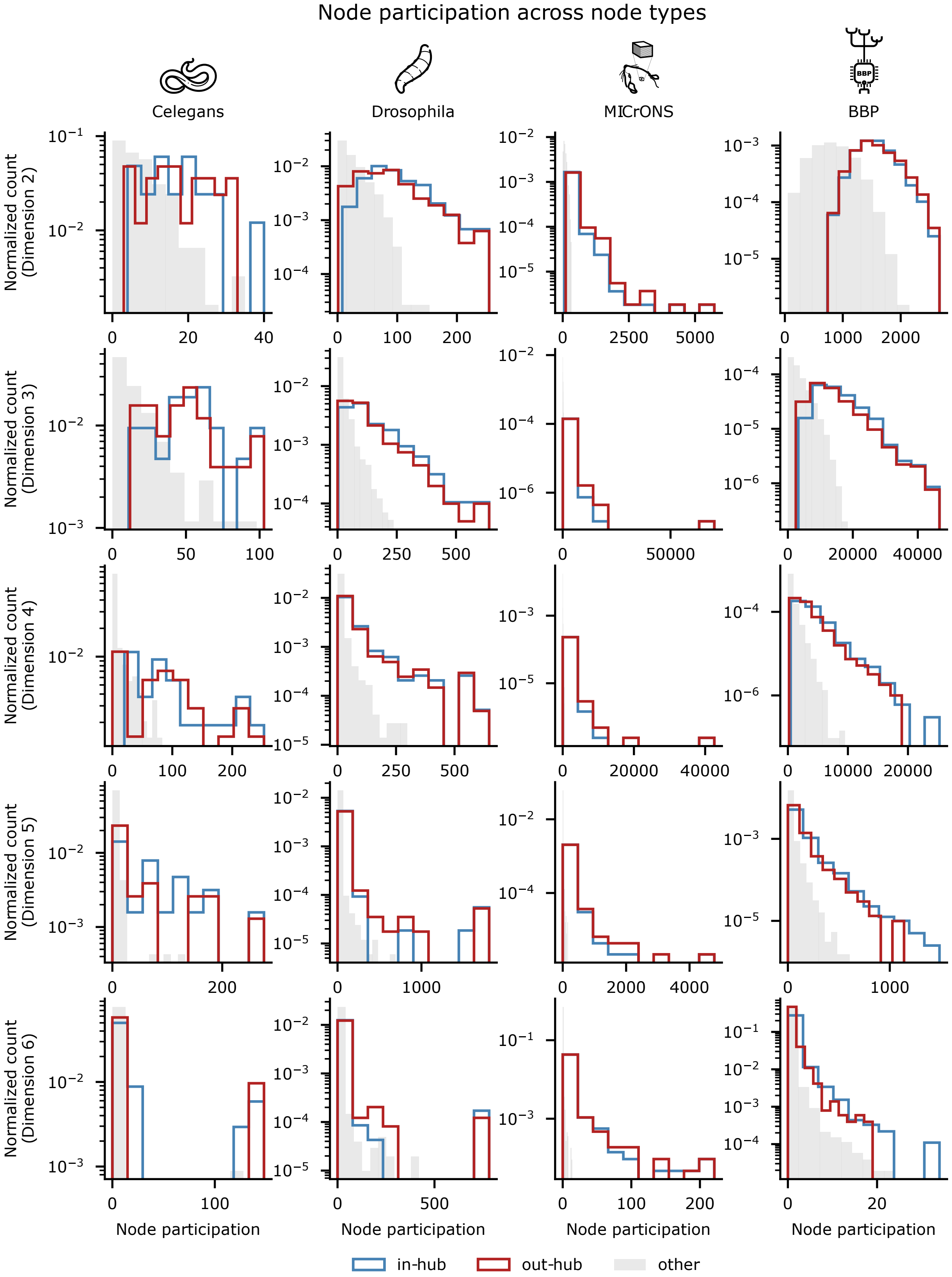

Figure S1: Node participation and network hubs, supplement to Figure 2. Normalized histograms of k-node participation for: all out-hubs in red, all in-hubs in blue, all other nodes in gray. In and out hubs were determined to be those in the top 10% of the in and out degree distributions. Rows correspond to the dimension of node participation $3 \leq k \leq 6$ and columns correspond to the four connectomes.

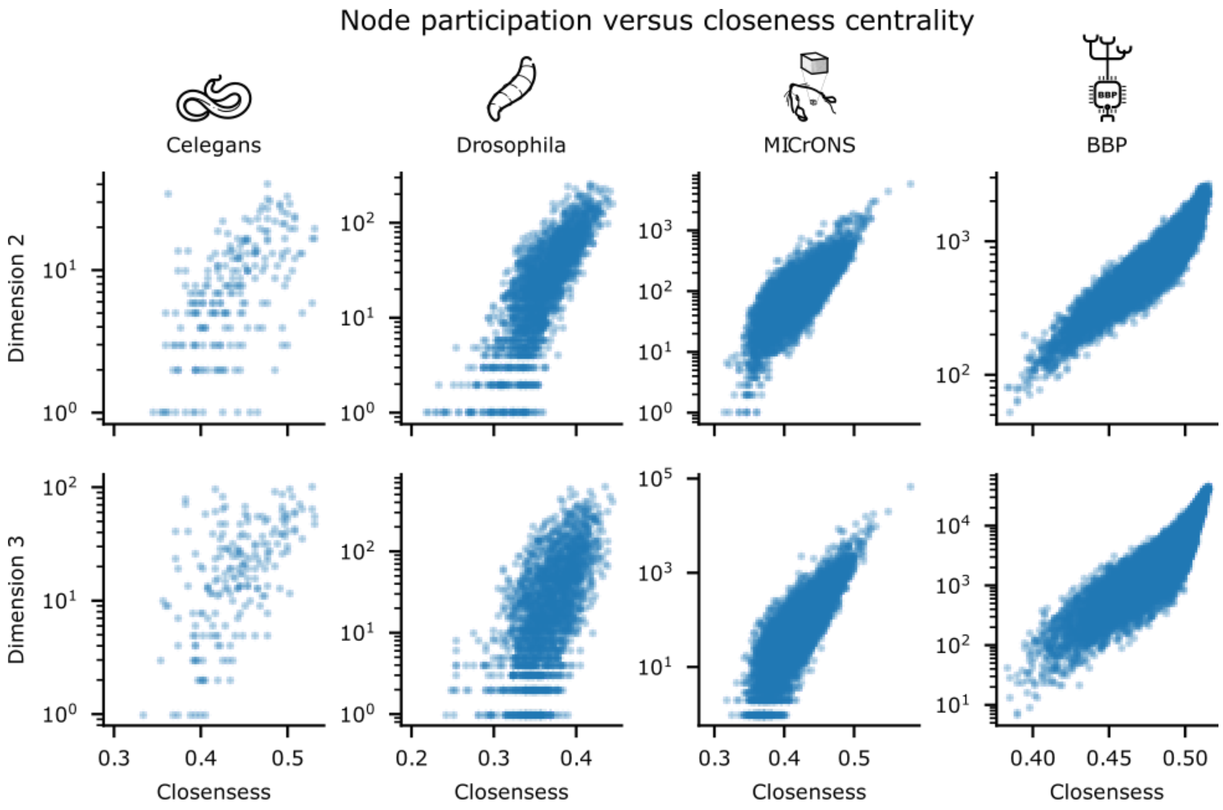

Figure S2: Node participation vs closeness centrality, supplement to Figure 2. Scatter plots of k-node participation (in the lower dimensions) in logarithmic scale versus closeness centrality. Rows correspond to the dimension of node participation $k = 2, 3$ and columns correspond to the four connectomes.

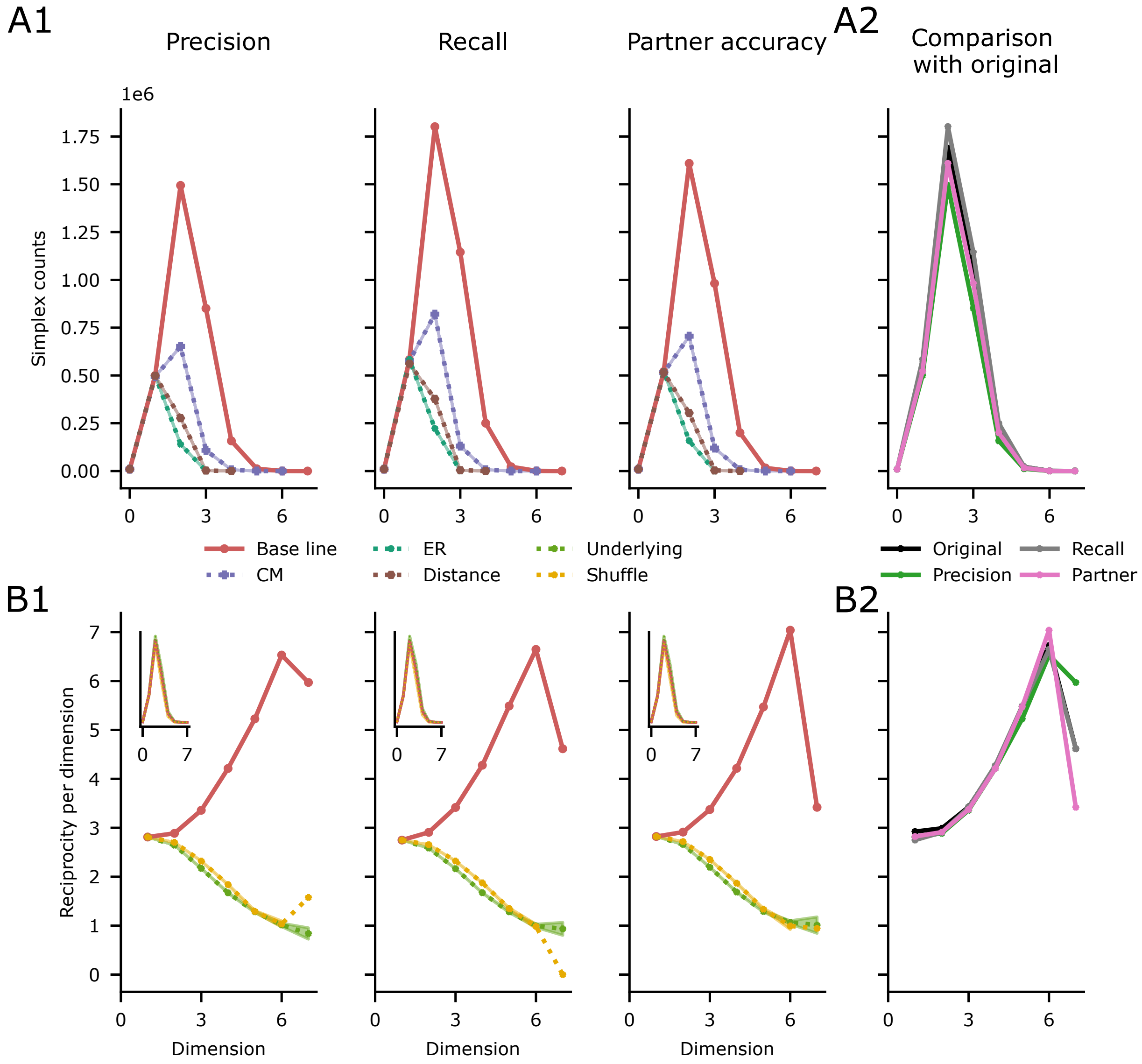

Figure S3: Assessing the effect of automatic detection in the MICrONS data set, supplement to Figure 2. Control connectomes were built to address address: precision (by removing 4% of the connections), recall (by adding 12% of connections) and partner accuracy (by shuffling 2% of the connections). Panels A1 and B1 reconstruct panels C and D of Figure 2. **A1:** Overexpression of simplex motifs with respect to 10 random controls of each type, which by design match the counts of the original network for dimensions 0 and 1. All counts in dimensions greater than one are significantly higher than the mean of the controls with p-values under 3×10^{-60} for a one sided one sample t-test. **A2:** Simplex counts for the original MICrONS connectome and the precision, recall and partner accuracy controls. **B1:** Percentage of reciprocal connections in the subgraph of simplices of each dimension contrasted with the same curve for controls where just the directionality of the connections is modified (see Methods). Inset: Simplex counts of the directional controls are close to the original ones. **B2:** Percentage of reciprocal connections on the subraphgs of simplices of the original MICrONS connectome and the precision, recall and partner accuracy controls.

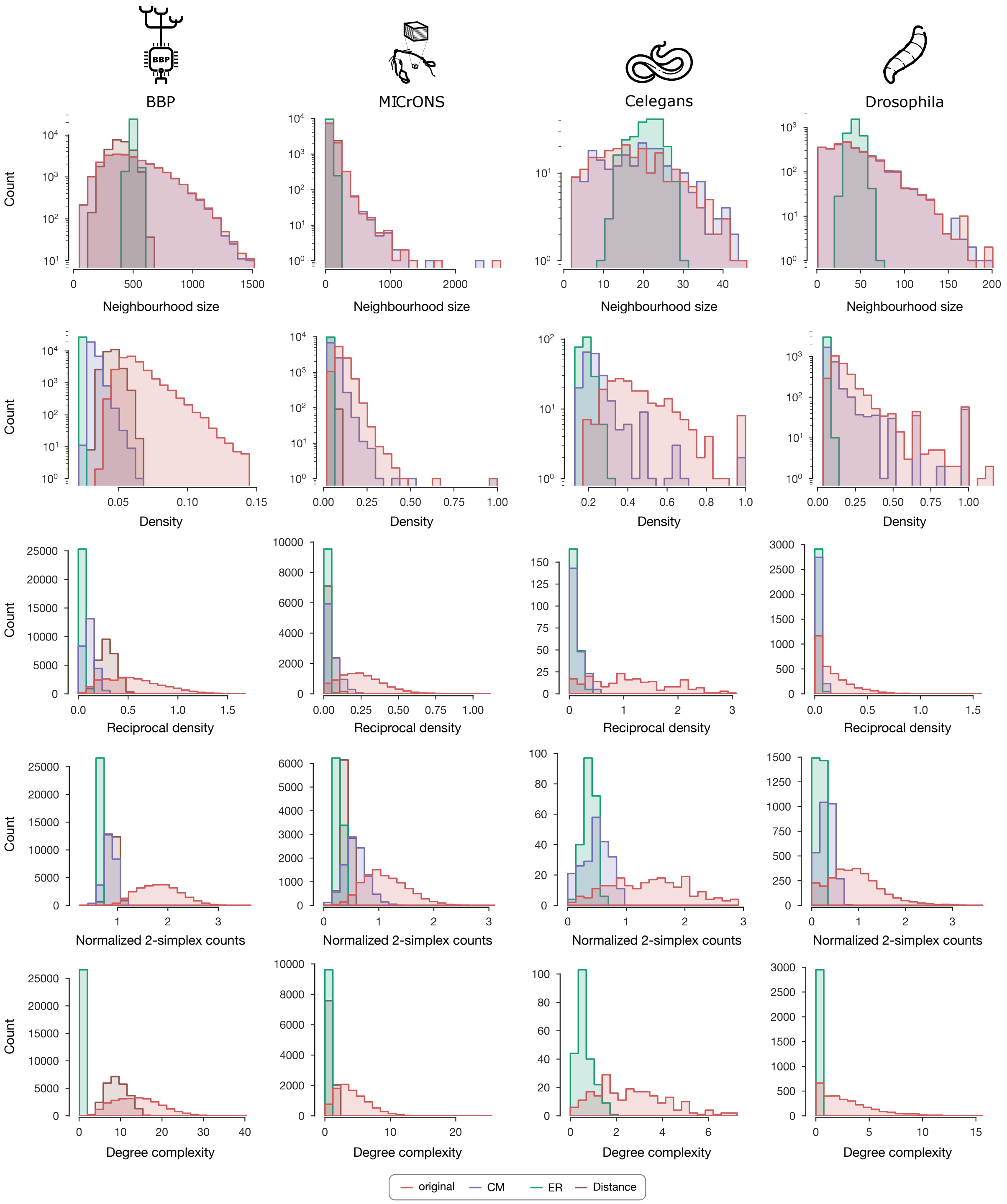

Figure S4: Network complexity promotes diversity of neighborhoods, supplement to Figure 3. Distribution of network metrics across neighborhoods for the original connectome and ER, CM and distance controls (when available). Row-by-row: Neighborhood size, density, reciprocal density, normalized 2-simplex counts, and degree complexity across connectomes. Normalization of 2-simplex counts was done by 1-simplex counts.

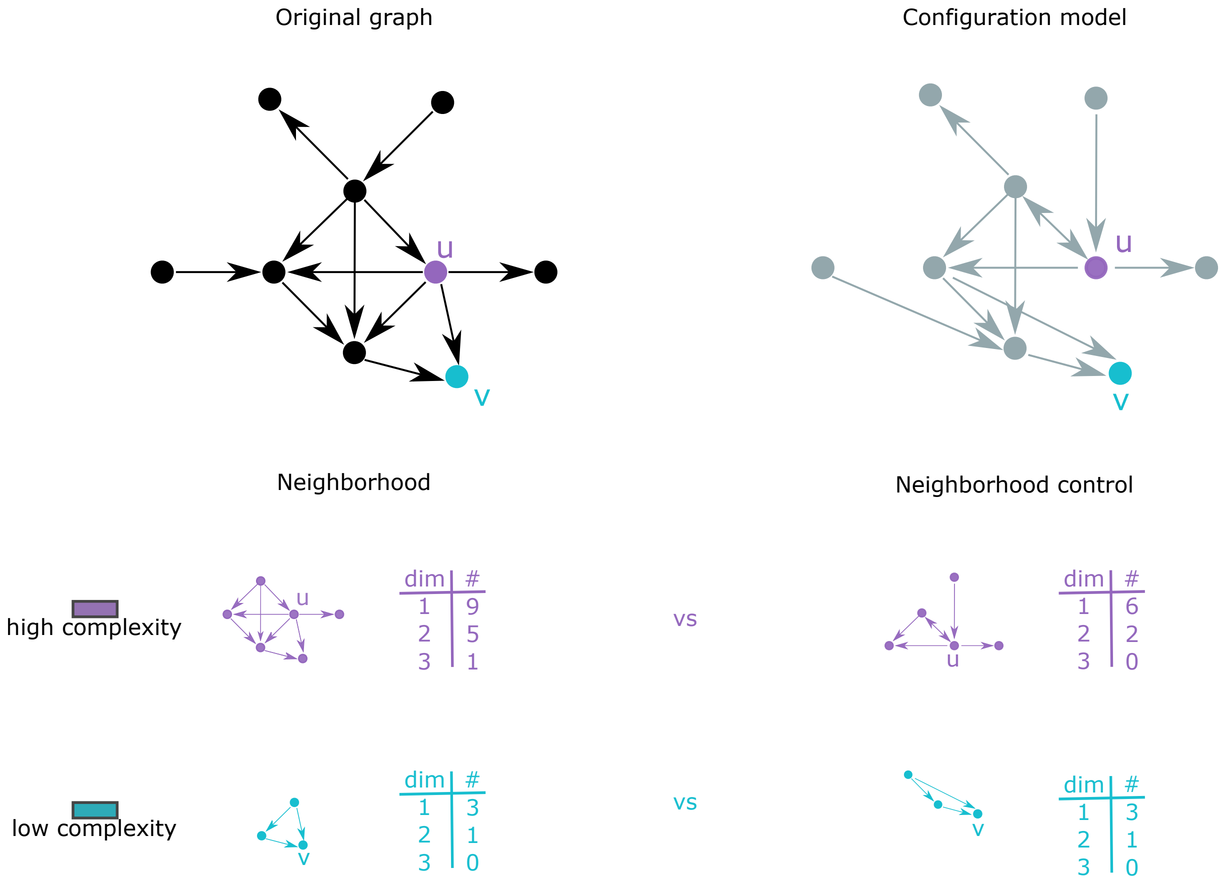

Figure S5: Illustrative example of simplicial/degree complexity, supplement to Figure 3 and STAR Methods-Network complexity. Top: Original base graph on the left and its corresponding control on the right. Bottom: The neighborhoods of the nodes u and v on the left and their corresponding controls on the right. The tables indicate the simplex counts in each dimension of each of the neighborhood subgraphs.

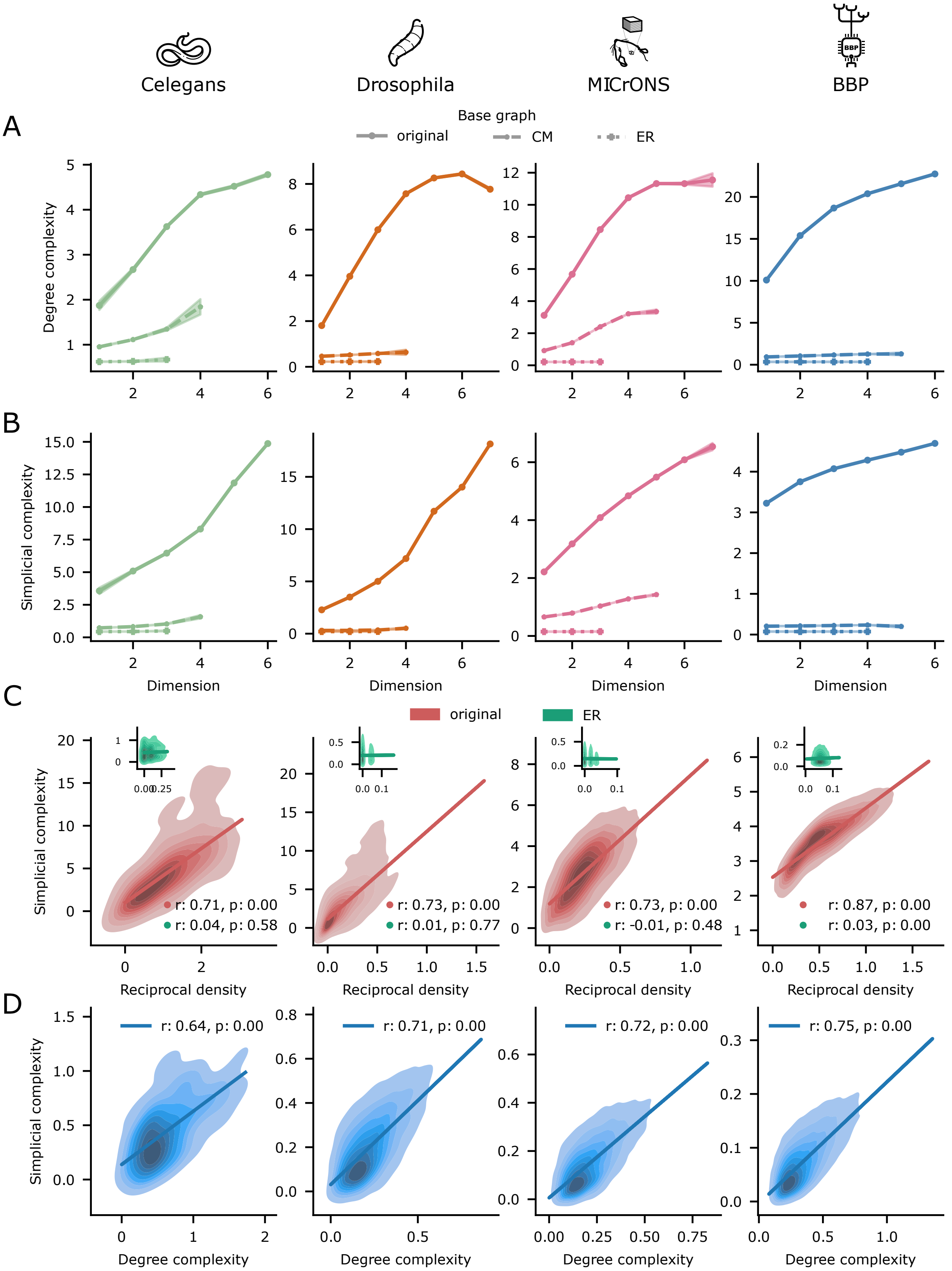

Figure S6: Neighborhood complexity (simplicial and degree), supplement to Figure 3. A: Average of neighborhood degree complexity on nodes that are on simplices of a given dimension(see Methods); drawn with solid, dashed and dotted lines for the original connectome a CM control and an ER control respectively. **B:** As A but with simplicial complexity instead. **C:** In BNNs reciprocal density is strongly correlated with simplicial complexity. Inset, this is not the case for ER graphs. **D:** The metrics of degree complexity and simplicial complexity are strongly correlated.

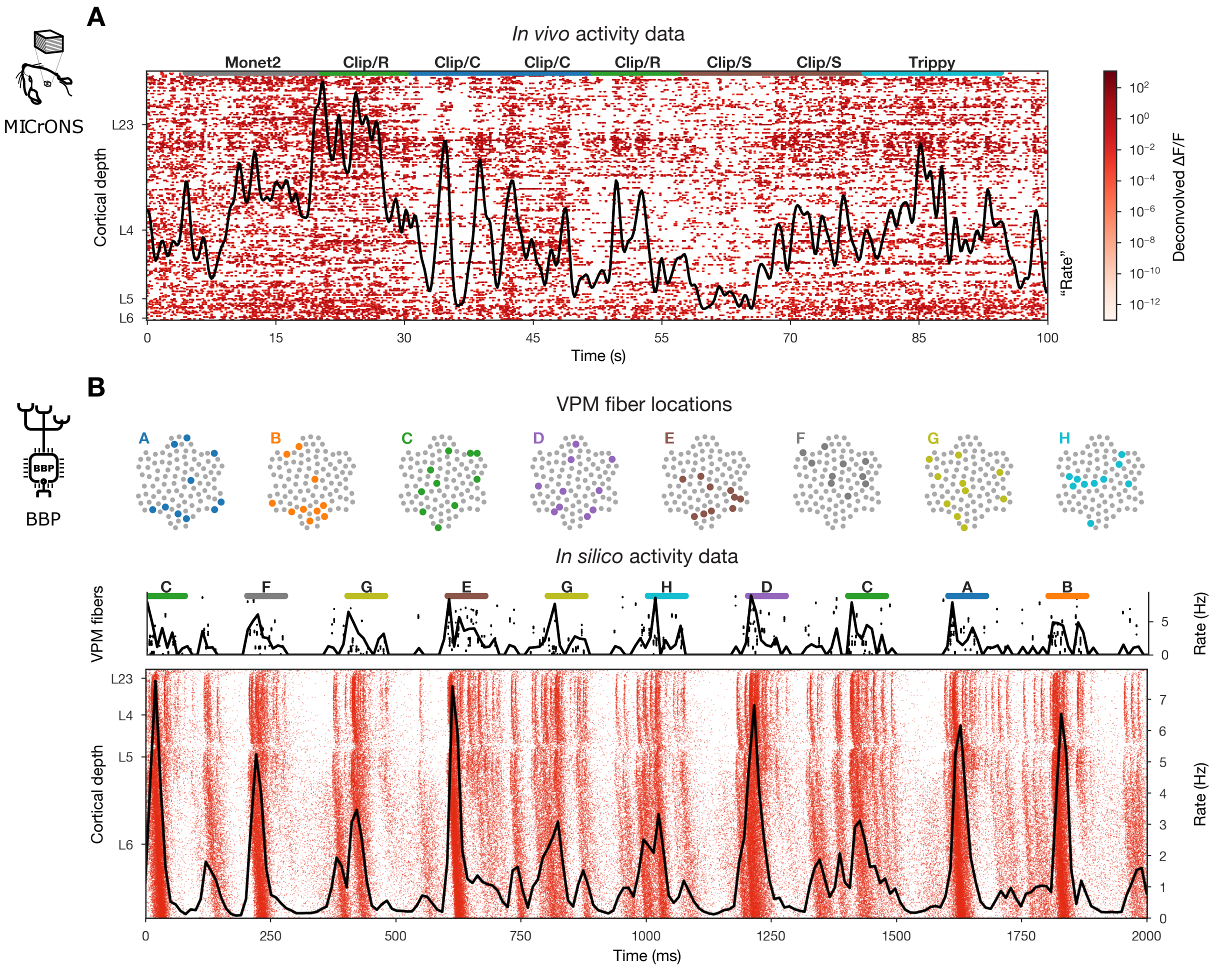

Figure S7: Activity datasets, supplement to Figure 4. Network activity and input stimuli on top for MICrONS (A) and BBP (B). **A:** MICrONS data shows deconvolved fluorescent traces, i.e., spike traces as extracted by the original authors[2]. It is 100 seconds from scan 7 from recording session 4. **B:** VPM input fiber bundles corresponding to the eight input patterns and input raster below. Raster plot (i.e., dots represent spikes from excitatory cells) of the BBP network.

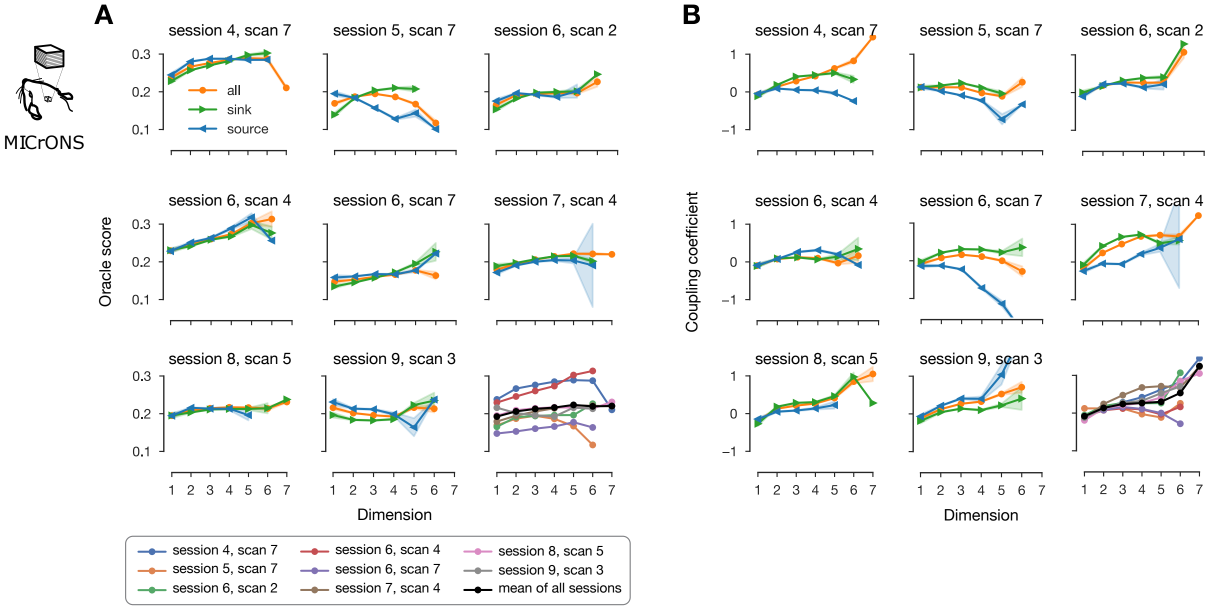

Figure S8: Per-scan activity metrics for MICrONS, supplement to Figures 4 and 6. Oracle score on panel **A** and coupling coefficient on panel **B** across simplices for the eight different recording sessions used (see Methods). The orange curves are the weighted averages of all neurons in that dimension, while the blue and green curves consider only the neurons in source or sink positions respectively. On the last subfigure, the curves for all nodes are pooled together across scans and the black line indicates the mean across sessions. (Mean across sessions is not the same as on the main figures, where the functional data was first z-scored and pooled together before running the analysis.) The same colors and legends as in A apply to B.

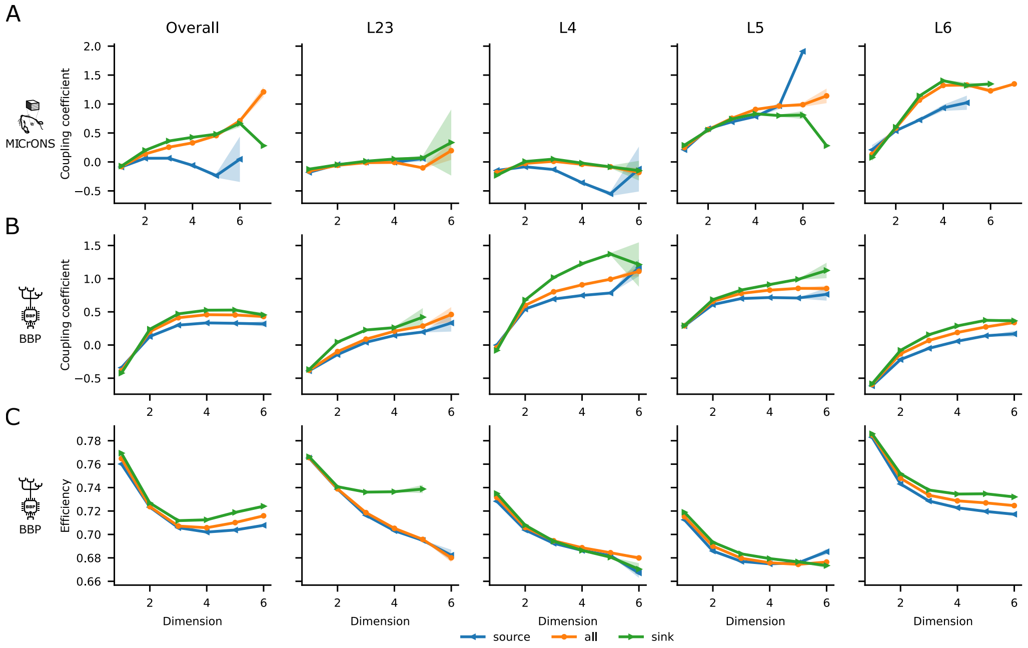

Figure S9: Coupling coefficient and efficiency across simplices split by layer, supplement to Figure 4. Coupling coefficient across simplices on panel **A** for MICrONS and **B** for BBP. On the left for all the neurons as in Figure 4A and on the right of it split across layers. **C:** As for **A** and **B** but for neighborhood efficiency (BBP data only). For all panels, the orange curves are the weighted averages of all neurons in that dimension, while the blue and green curves consider only the neurons in source or sink positions respectively.

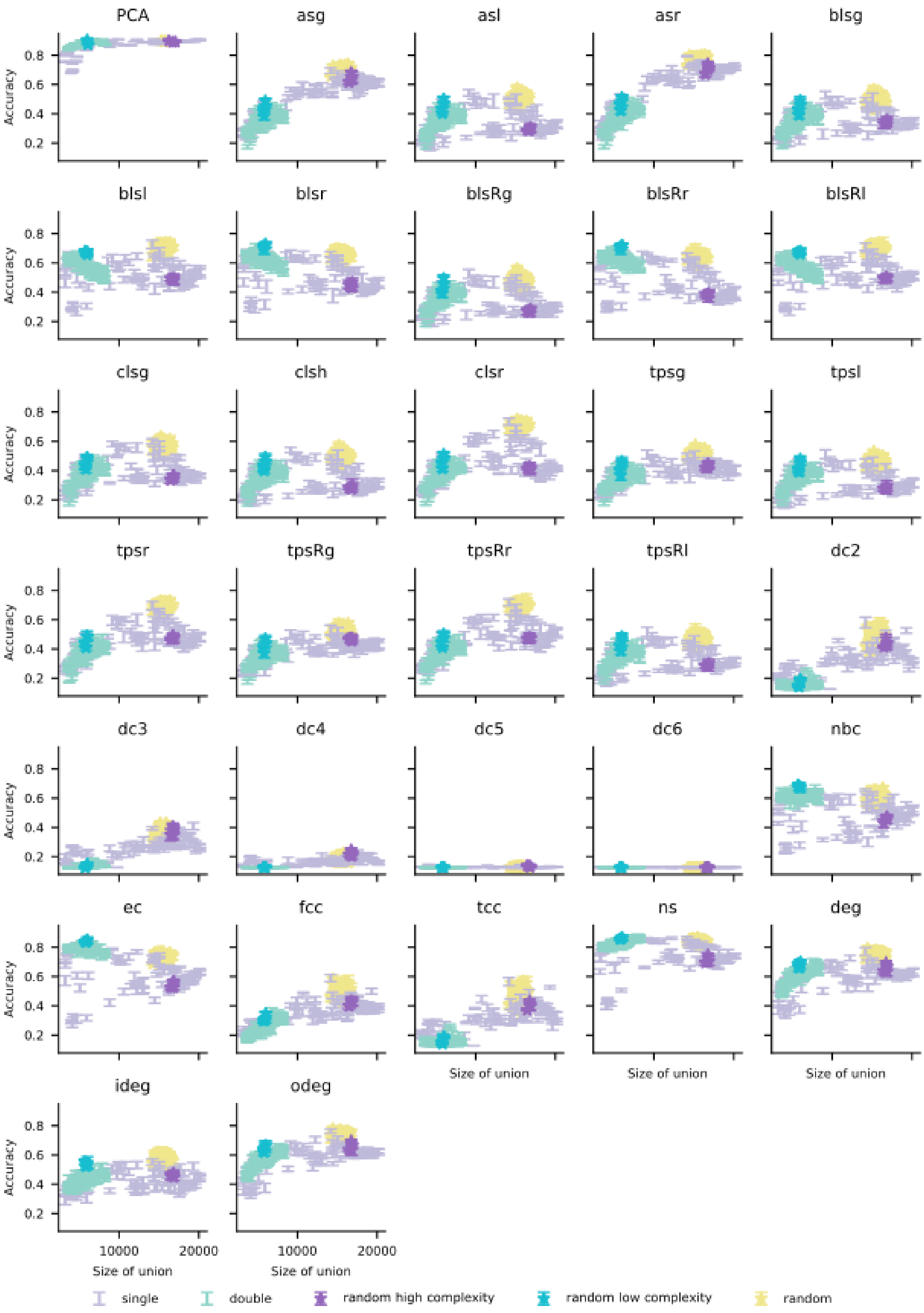

Figure S10: Classification accuracy for a given population size, supplement to Figure 4. Classification accuracy for all feature parameters. Each panel corresponds to a feature parameter, which is indicated in its title. Within each panel each bar corresponds to a selection of 50 neighborhoods selected either at random or maximizing/minimizing a given selection parameter. The x-axis of the bars corresponds to the size of the union of the 50 neighborhoods selected. Its center in the y-axis corresponds to the the accuracy for that (selection,feature) pair and the height of the bars is given by the cross validated error. Both selection and featurization parameters and their abbreviations are listed in Table S1.

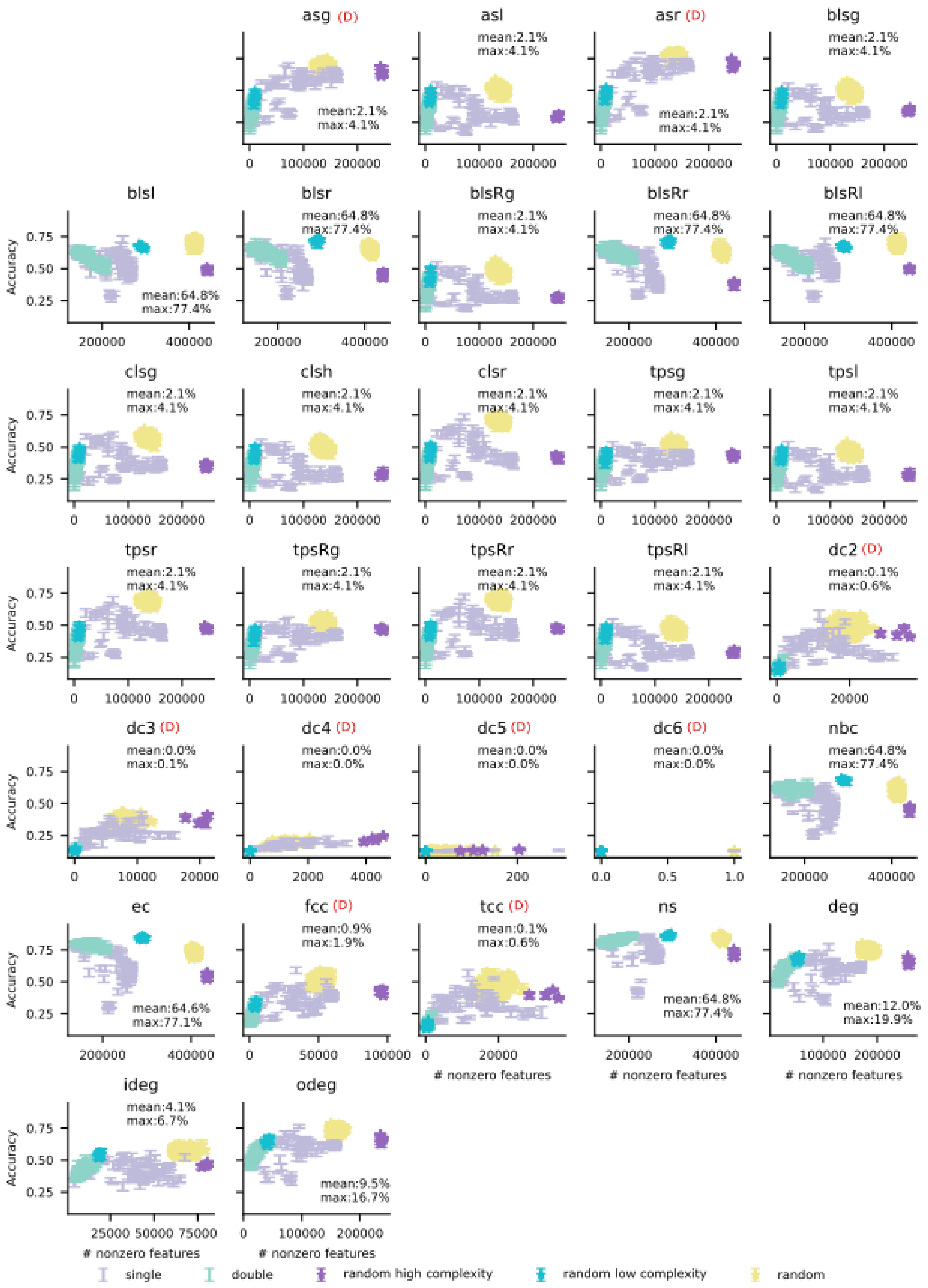

Figure S11: Classification accuracy versus number of nonzero features, supplement to Figure 4. Classification accuracy for all feature parameters as for Fig. S10 but where x-axis is given by the number of non-zero features in the featurization vector of that (selection,feature) pair. The legend indicates the mean and maximum percentage of nonzero entries in the feature vectors for the double classification procedure across all selection parameters. Degenerate featurization parameters (those for which most of the entries of their feature vectors for double selection are 0) have a (D) marking in their corresponding panels.

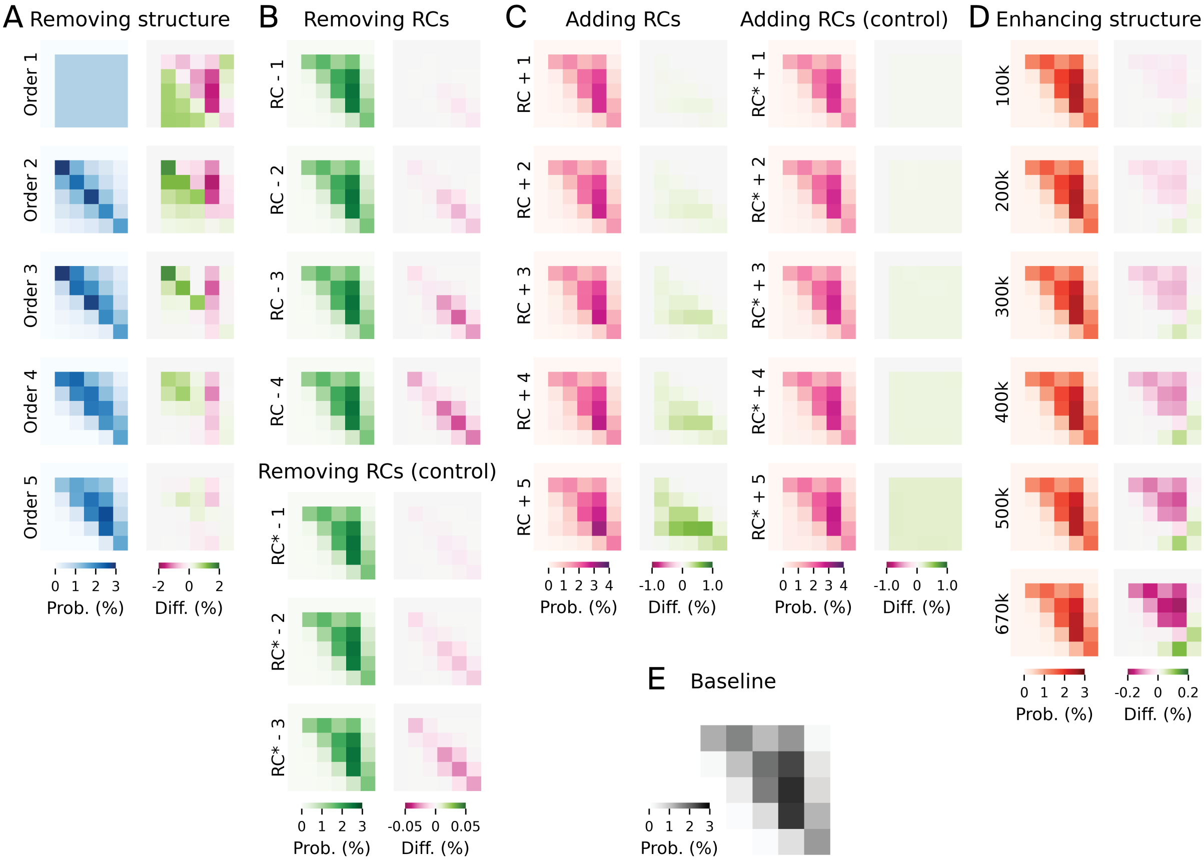

Figure S12: Structural validation of manipulated connectomes, supplement to Figure 5. For all manipulated connectomes shown in Fig. 5 and summarized in Table 2, the layer-wise connection probabilities when removing structure (**A**), removing reciprocal connections (**B**), adding reciprocal connections (**C**), and enhancing structure (**D**), and their differences to the baseline connectome (**E**) are illustrated. Asterisks indicate random controls. In the first column of each panel, the mean connection probabilities between excitatory neurons in each pre-synaptic (y axis) and post-synaptic (x axis) layer are shown. Note that layer 1 is empty. In the second column, the differences to the original connectome are highlighted. Note the different color scales at the bottom.

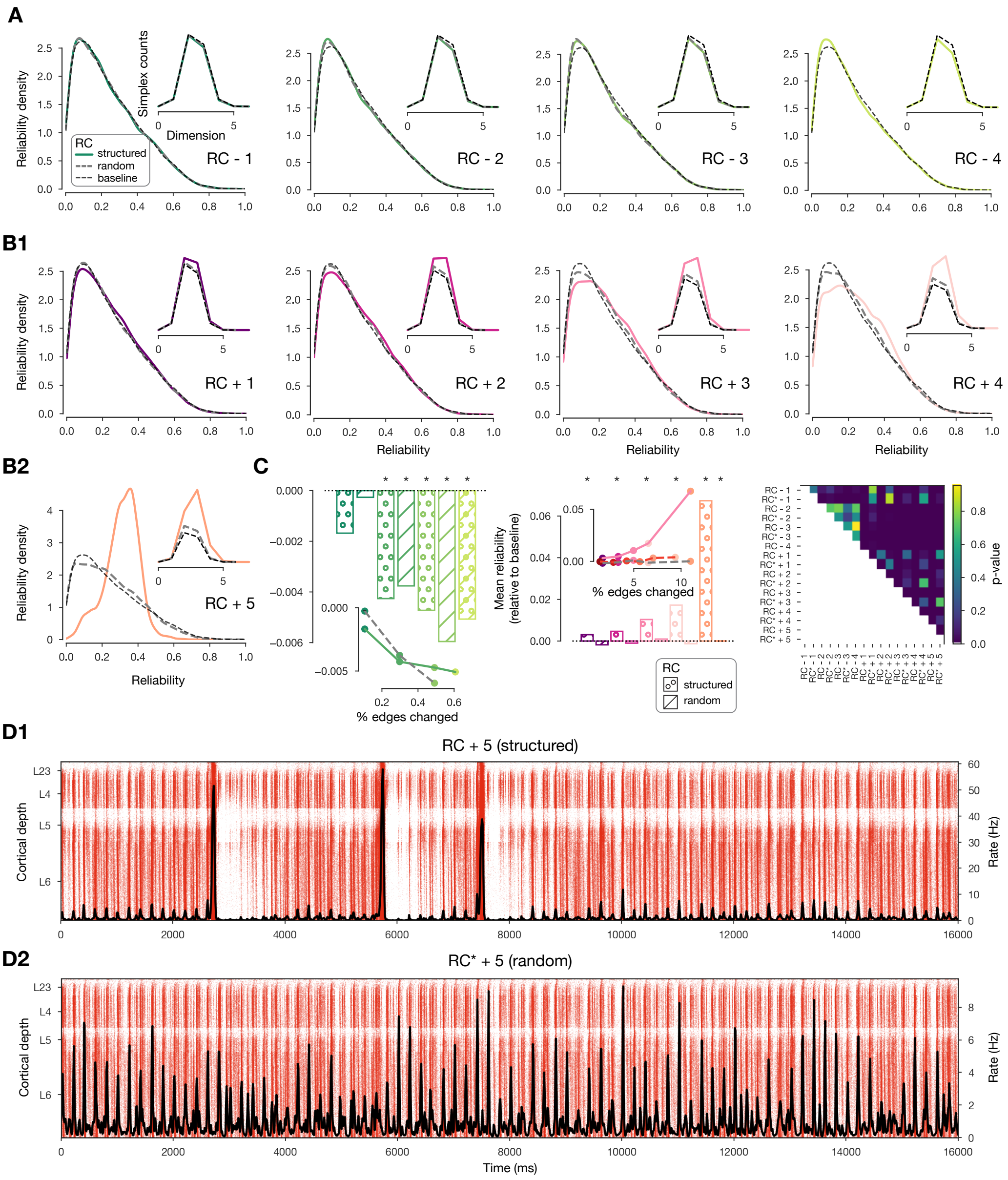

Figure S13: Reliability of manipulated connectomes relative to their controls, supplement to Figure 5. Distributions of neurons in a manipulated connectome (color), its control (dashed grey) and the baseline connectome (dashed black). Insets show corresponding simplex counts with the same color coding. Panel **A** shows manipulations where reciprocal connections are removed and their controls i.e., $RC-k$ and $RC*-k$. See Table 2 for details. Panel **B** as A but for reciprocal connections added i.e., $RC+k$ and $RC*+k$. **C:** Left change in mean reliability relative to the baseline connectome for reciprocal connections removed. The bars marked with a $*$ indicate when the change is statistically significant according to a KW-test. The insets show the change in mean reliability vs % of the edges that have been changed. Middle, as left but for reciprocal connections added. Right, p-values of the KW-test between all manipulated connectomes in the Figure. **D1, D2**: Raster plot of the activity in $RC+5$ and $RC*+5$ respectively, showing that $RC+5$ has transitioned to synchronous activity.

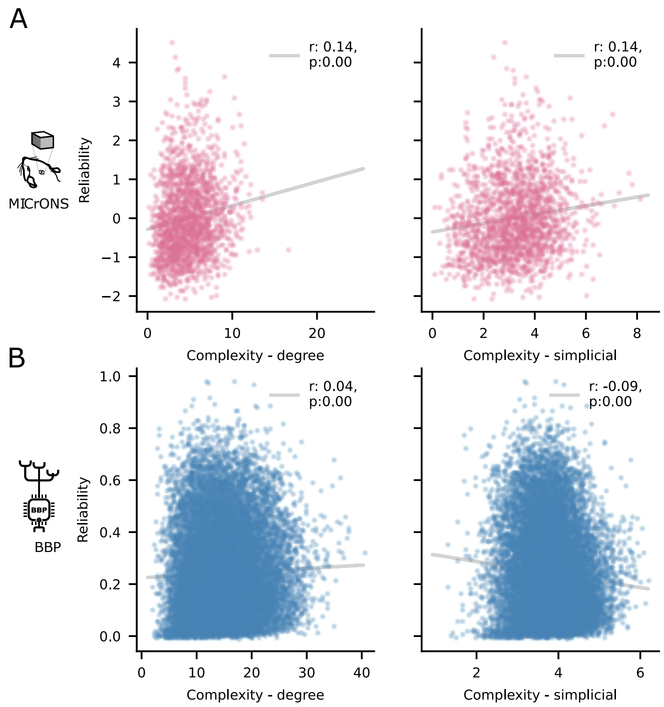

Figure S14: Reliability and neighborhood complexity, supplement to Figure 6. Negligible correlation is found between the neighborhood complexity and the reliability of its center, both for the degree and simplicial complexity metrics.

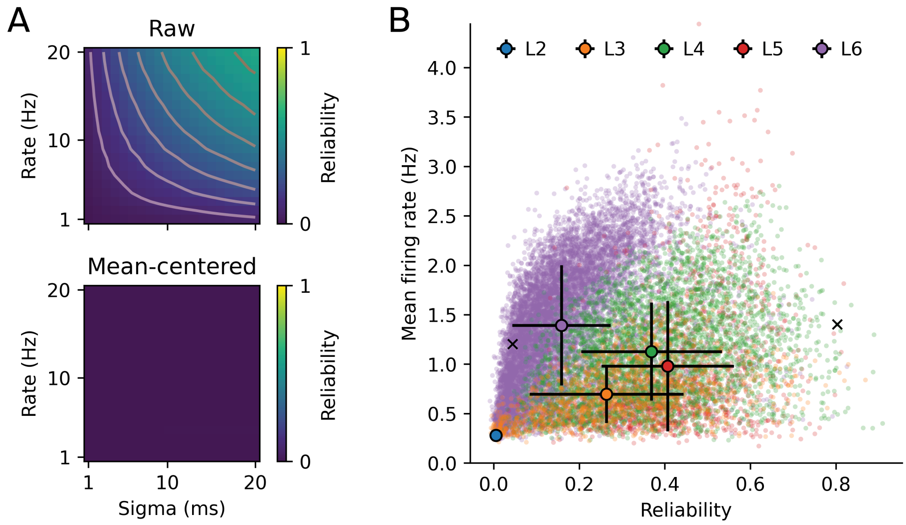

Figure S15: Reliability versus firing rates, supplement to STAR Methods-Reliability. A: Gaussian kernel reliability values of randomly generated Poisson spike trains with different firing rates and computed with different values of sigma. Without mean-centering of the data, there is a clear dependence of reliability on firing rates (upper panel) which disappears completely when mean-centering the data (lower panel). **B:** Mean-centered Gaussian kernel reliability values ($\sigma = 10\,\text{ms}$) versus mean firing rates of all excitatory neurons in BBP data, colored by layer. Error bars denote mean $\pm$ SD per layer. $\times$ markers indicate neurons with example spike trains shown in Fig. 1A.

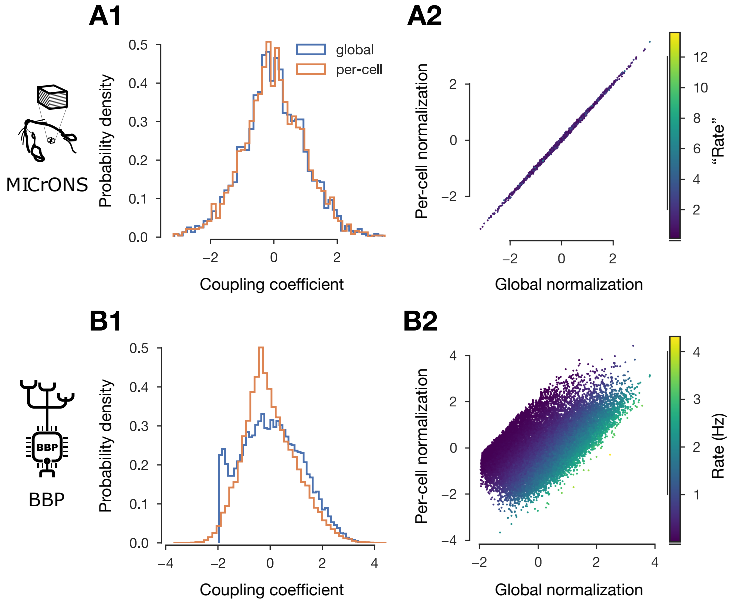

Figure S16: Normalization of raw coupling coefficients, supplement to STAR Methods-Coupling coefficient. A1 and **B1:** Global vs. stricter per-cell (keeping the firing rate, see Methods) normalization of coupling coefficients for MICrONS (A1) and BBP (B1). **A2** and **B2:** Correlation between global and per-cell normalized values and their link to firing rate (especially for BBP, where the two distributions differ.).

References

[1] Pedro Conceição, Dejan Govc, Jānis Lazovskis, Ran Levi, Henri Riihimäki, and Jason P Smith. An application of neighbourhoods in digraphs to the classification of binary dynamics. *Network Neuroscience*, 6(2):528–551, 2022.

[2] MICrONS Consortium, J Alexander Bae, Mahaly Baptiste, Caitlyn A Bishop, Agnes L Bodor, Derrick Brittain, JoAnn Buchanan, Daniel J Bumbarger, Manuel A Castro, Brendan Celii, et al. Functional connectomics spanning multiple areas of mouse visual cortex. *bioRxiv*, pages 2021–07, 2021. doi: 10.1101/2021.07.28.454025.
